# Nano-modulators with the function of disrupting mitochondrial Ca^2+^ homeostasis and photothermal conversion for synergistic breast cancer therapy

**DOI:** 10.1186/s12951-023-02220-7

**Published:** 2023-12-04

**Authors:** Chenglong Wang, Tao Li, Zhen Wang, Yao Li, Yan Liu, Maochang Xu, Zongquan Zhang, Yiping Deng, Liang Cai, Chunxiang Zhang, Chunhong Li

**Affiliations:** 1 Department of Pharmaceutical Sciences, School of Pharmacy, Southwest Medical University, 1-1 Xianglin Road, Luzhou, Sichuan 646000 People’s Republic of China; 2 Key Laboratory of Medical Electrophysiology of Ministry of Education, Institute of Cardiovascular Research, Southwest Medical University, Sichuan Province, Luzhou, China; 3 Department of Science and Technology, Southwest Medical University, Luzhou, China; 4 Nuclear Medicine Department of the First Affiliated Hospital, Southwest Medical University, Luzhou, 646000 Sichuan China; 5 The Key Laboratory of Medical Electrophysiology of the Ministry of Education, Southwest Medical University, No.1, Section 1, Xianglin Road, Luzhou, Sichuan 646000 People’s Republic of China

**Keywords:** Mitochondrial Ca^2+^ overload, Photothermal therapy, Breast cancer, Curcumin, Indocyanine green

## Abstract

Breast cancer treatment has been a global puzzle, and apoptosis strategies based on mitochondrial Ca^2+^ overload have attracted extensive attention. However, various limitations of current Ca^2+^ nanogenerators make it difficult to maintain effective Ca^2+^ overload concentrations. Here, we constructed a multimodal Ca^2+^ nano-modulator that, for the first time, combined photothermal therapy (PTT) and mitochondrial Ca^2+^ overload strategies to inhibit tumor development. By crosslinking sodium alginate (SA) on the surface of calcium carbonate (CaCO_3_) nanoparticles encapsulating with Cur and ICG, we prepared a synergistic Ca^2+^ nano-regulator SA/Cur@CaCO_3_-ICG (SCCI). In vitro studies have shown that SCCI further enhanced photostability while preserving the optical properties of ICG. After uptake by tumor cells, SCCI can reduce mitochondrial membrane potential and down-regulate ATP production by producing large amounts of Ca^2+^ at low pH. Near-infrared light radiation (NIR) laser irradiation made the tumor cells heat up sharply, which not only accelerated the decomposition of CaCO_3_, but also produced large amounts of reactive oxygen species (ROS) followed by cell apoptosis. In vivo studies have revealed that the Ca^2+^ nano-regulators had excellent targeting, biocompatibility, and anti-tumor effects, which can significantly inhibit the proliferation of tumor cells and play a direct killing effect. These findings indicated that therapeutic strategies based on ionic interference and PTT had great therapeutic potential, providing new insights into antitumor therapy.

**Graphical Abstract:**

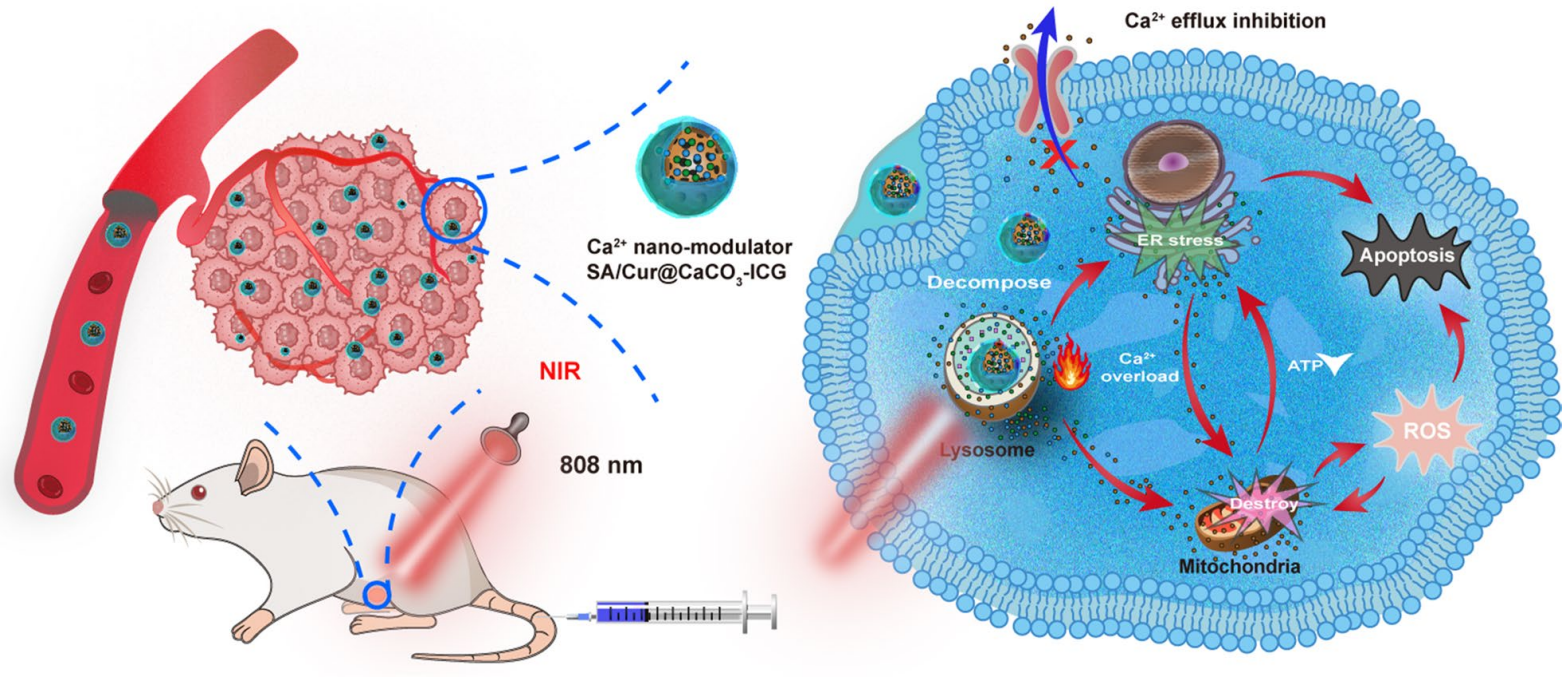

**Supplementary Information:**

The online version contains supplementary material available at 10.1186/s12951-023-02220-7.

## Introduction

Breast cancer is a high incidence cancer in women that involves the development of lobules, ducts, or connective tissue in the breast [1, 2]. Traditional cancer chemotherapy is highly efficient and has unique advantages, but its clinical efficacy is limited due to the complexity, variability and drug resistance of tumors. Therefore, there is an urgent need to develop effective and rational treatment options to address the world's breast cancer treatment dilemma. It is well-known that mitochondria are not only the dynamical compartment of eukaryotic cells, but also a prominent signaling platform that regulates cell metabolism, proliferation, death, and immune response [3–6]. In fact, tumor cells prevent apoptosis by altering mitochondrial metabolism and signaling pathways [7, 8]. As a result, inducing persistent mitochondrial dysfunction in tumor cells is an effective way to kill cancer cells. So far, cancer treatment strategies based on inducing mitochondrial apoptosis have emerged in an endless stream, including reducing mitochondrial membrane potential (MMP) [9], increasing mitochondrial reactive oxygen species (ROS) generation [10], mitochondrial DNA stress [11], and Ca^2+^ overload to cut off the supply of ATP to tumor cells by activating caspase-dependent apoptosis [12, 13].

Mitochondrial Ca^2+^ overload is an effective method to induce apoptosis in tumor cells [14–16]. Currently, intracellular Ca^2+^ nano-modulators based on tumor microenvironment responses such as calcium carbonate (CaCO_3_) and calcium phosphate have been widely developed for Ca^2+^ overloading mediated cancer therapy [17–20]. By disrupting mitochondrial Ca^2+^ homeostasis, mitochondrial morphology changes, membrane potential is reduced, and functional disorders are induced [21–23]. Calcium carbonate nanoparticles are excellent Ca^2+^ release carriers with excellent biocompatibility and biodegradability [24–26], and can provide a steady stream of Ca^2+^ to tumor cells. However, the continuous excretion of intracellular Ca^2+^ reduces the anticancer effect due to the presence of Ca^2+^ channels [27, 28]. Tumor cells are more sensitive to increases in Ca^2+^ concentration than normal cells, and changes in Ca^2+^ signaling pathways can further promote Ca^2+^ overload in mitochondria [19, 29]. Bioactive drugs such as curcumin (Cur) can not only inhibit the efflux of Ca^2+^ from the cytoplasm to the extracellular matrix, but also promote the release of Ca^2+^ in the endoplasmic reticulum (ER), greatly increasing the concentration of Ca^2+^ in the cytoplasm [30, 31].

It is necessary to combine chemotherapy with alternative therapies such as immunotherapy, photodynamic therapy (PDT) and photothermal therapy (PTT) to eradicate or maximize the inhibition of tumor growth [32, 33]. PTT is an accurate and efficient non-invasive method for tumor ablation, with the advantages of minimal invasion and minimal damage to normal cells [34]. Indocyanine green (ICG), a fluorescent dye capable of absorbing near-infrared light radiation (NIR) laser with wavelength of 808 nm, is approved by the U.S. Food and Drug Administration for clinical biological imaging and disease diagnosis [35, 36]. Under the action of single-wavelength NIR laser, ICG is able to simultaneously achieve the combined treatment of PTT and PDT, generating ROS and increasing temperature in the tumor [37, 38]. However, as a free agent, ICG is unstable, easy to decompose, lack of targeting, fast clearance rate, and low cell uptake, which limits the efficacy of PDT/PTT [39, 40]. CaCO_3_ is a natural and non-toxic inorganic biomaterial with a variety of intrinsic advantages. These limitations can be minimized by encapsulating ICG into CaCO_3_ nanoparticles. Sodium alginate (SA) is a natural polysaccharide derived from kelp or Sargassum algae of brown algae. Extensive research on the physical and chemical properties of SA demonstrates that it is an essential biological material with non-toxicity, high biocompatibility and excellent biodegradability [41]. Most importantly, in the presence of multivalent cations (Ca^2+^, Zn^2+^, Mg^2+^), alginate is prone to cross-link to form gels, which has significant application value in drug delivery, tissue repair, and 3D bioprinting [42, 43]. As a qualified goalkeeper, SA realizes the coating of CaCO_3_ nanoparticles through Ca^2+^ crosslinking to reduce their hydrophobicity, prolong the blood circulation and reduce the leakage of drug molecules.

**Fig. 1:**
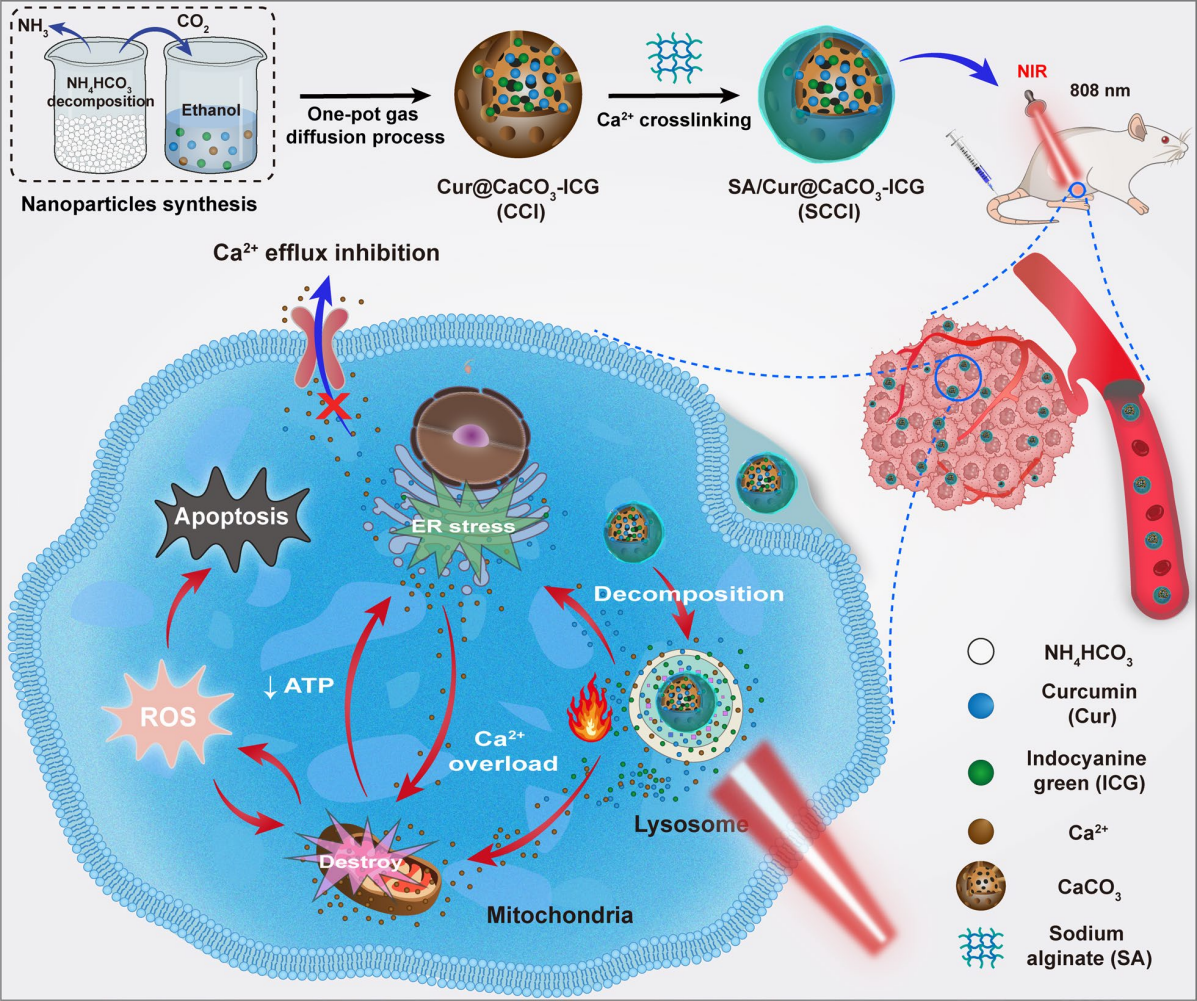
Schematic diagram of the synthesis of SCCI and the mechanism of its mediated mitochondrial Ca^2+^ overload and PTT synergistic cascade cancer therapy

Here, we constructed a multimodal Ca^2+^ nano-modulator, SA/Cur@CaCO_3_-ICG (SCCI), which can efficiently induce tumor cell apoptosis or directly kill tumor cells through a combination of chemotherapy, PTT, and mitochondrial Ca^2+^ overload (Fig. 1). After entering into tumor cells, the Ca^2+^ nano-modulators achieved lysosome escape by consuming H^+^, while releasing large amounts of Ca^2+^ and Cur. The presence of Cur promoted the release of Ca^2+^ from the ER to the cytoplasm and suppressed the efflux of Ca^2+^, exacerbating the tumor cell apoptosis induced by the imbalance in mitochondrial homeostasis. Additional laser irradiation can further accelerate the decomposition of CaCO_3_, promote the production of large amounts of ROS by the ICG, and significantly increase the tumor site temperature to directly kill tumor cells. Overall, the multimodal Ca^2+^ nano-modulators provided a promising strategy for multivariate combination therapy in breast cancer.

## Experimental section

### Materials

Calcium chloride (CaCl_2_) anhydrous was purchased from Macklin (Shanghai, China). Ammonium bicarbonate (NH_4_HCO_3_) was obtained from Tianjin Chemical Three Plant (Tianjin, China). Curcumin (Cur) was obtained from Shanghai Yuanye Bio-Technology (Shanghai, China). Indocyanine green (ICG) was purchased from Meryer (Shanghai, China). Methyl thiazolyl tetrazolium (MTT) was purchased from BioFroxx (Guangzhou, China). Sodium alginate (SA), rhodamine B (RhB), 2′,7′-dichlorofluorescein diacetate (DCFH-DA), 2-(4-amidinophenyl)-6-indolecarbamidine dihydrochloride (DAPI) and Fluo-3, AM were purchased from Solarbio Science & Technology (Beijing, China). Calcein-AM/PI kit, ATP detection kit and MMP assay kit with JC-1were purchased from Solarbio Science & Technology (Beijing, China). Coumarin 6 (C6) was obtained from Macklin (Shanghai, China). Lyso-Tracker Red was purchased from Beyotime (Shanghai, China).

### Cell lines and animals

4T1 cells were cultured in RPMI-1640 medium containing 10% (v/v) fetal bovine serum and 1% (v/v) penicillin/streptomycin. The medium was then placed in a humidified incubator with 5% carbon dioxide (CO_2_) at 37 °C.

Female BALB/c mice came from GemPharmatech (Chengdu, China), and were kept under standard conditions. All animal experiments were approved by the Animal Care and Ethics Committee of Southwest Medical University. In situ mouse breast cancer model was established by injecting 5 × 10^5^ 4T1 cells into the right breast pad of BALB/c mice (6 weeks old). When the tumor volume was around 100 mm^3^, animal studies started. The tumor volume can be calculated using the following formula:

$$V=\frac{Length \times {(width)}^{2}}{2}$$

### Preparation of Cur@CaCO_3_-ICG (CCI) and SCCI nanoparticles

CCI and SCCI nanoparticles were synthesized by the one-pot gas diffusion process. In general, three beakers were placed in an airtight container. The first beaker contained 10.0 g of NH_4_HCO_3_, which was used to produce large amounts of CO_2_. 100.0 mL of ethanol dissolved with 50.0 mg CaCl_2_, 8.0 mg Cur and 5.0 mg ICG was added to another beaker and covered with a membrane with several holes. The CO_2_ produced by NH_4_HCO_3_ continually diffused into ethanol solution containing CaCl_2_, Cur, and ICG as a source of carbonate ions (CO_3_^2−^). Dissolved Ca^2+^ reacted with CO_3_^2−^ to form CaCO_3_ nanoparticles, enabling encapsulation of the above drugs. The last beaker was filled with 250.0 mL of deionized water to absorb the excess NH_3_ produced by NH_4_HCO_3_. After reaction at room temperature for 12 h, the collected CCI was repeatedly centrifuged at 10,000 rpm and washed twice with ethanol. The prepared nanoparticles were added into SA dissolved deionized water and stirred at room temperature for 2 h. The mass ratio of CCI and SA was 1:10. The collected SCCI nanoparticles were centrifuged twice at 10,000 rpm and washed with deionized water. Finally, the nanoparticles were freeze-dried for further use.

### Characterization of nanoparticles

The morphology of CCI and SCCI were characterized by transmission electron microscopy (TEM, JEM 1200EX; JEOL, Japan). In addition, the zeta potential of the preparations was further analyzed using a Malvern Zetasizer (Nano ZS90, Malvern Instruments, UK). Energy dispersive X-ray spectroscopy (EDX, FEI Tecnai G2 F30, USA) and X-ray photoelectron spectroscopy (XPS, Thermo escalab 250XI, America) were used to determine the element composition and visual distribution of SCCI. X-ray diffractometer (XRD, Brucker D8 Advance, Germany) was used to evaluate the crystal properties of the samples. The freeze-dried samples were scanned using a Fourier Transform infrared spectrometer (FT-IR, Nicolet 6700, USA) with a scanning range of 4000–500 cm^−1^. The optical properties of the nanoparticles were then characterized using an ultraviolet visible (UV–vis) spectrophotometer (Shimadzu UV-3600Plus, Japan), and the ICG loading efficiency (LE) was verified at 780 nm. The loading LE of ICG is calculated as follows:

$$LE\, of\, ICG \left(\%\right)=\frac{Weight\, of \,loading\, ICG}{Weight \,of \,feeding \,ICG} \times 100\%$$

The loading content (LC) and LE of Cur were determined by HPLC according to the following conditions. The mobile phase was mixed with 4% glacial acetic acid and acetonitrile (55:45, v/v). The flow rate was 1.0 mL/min, the detection wavelength was set to 426 nm, and the column temperature was 30 °C. The LC and LE for Cur are calculated as follows:

$$LC\, of\, Cur \left(\%\right)=\frac{Weight \,of \,loading \,Cur}{Total \,weight \,of \,nanoparticle} \times 100\%$$

$$LE \,of \,Cur \left(\%\right)=\frac{Weight \,of \,loading \,Cur}{Weight \,of \,feeding \,Cur} \times 100\%$$

### Hemolysis assay

Red blood cells (RBC) were obtained from BALB/c mice and diluted with saline to 2% suspension. Cur, ICG, CCI, and SCCI were added to 2% red blood cell suspension to the desired concentrations (5, 25, 50, and 200 μg/mL). It was then incubated at 37 °C for 3 h. An identical red cell suspension incubated with saline and ultrapure water was used as a negative and positive control under the same conditions. All samples were centrifuged at 3,000 rpm for 10 min before accurately absorbing the same volume of the supernatant into a 96-well plate. The hemoglobin release at 540 nm was measured by Varioskan Flash microplate reader.

### Acid-responsive release of Cur in the SCCI

The SCCI (2 mg/mL) was dispersed in PBS solution with pH 5.0, 6.5, and 7.4 for 1 h. The sample was centrifuged at 10,000 rpm for 10 min and the color depth and precipitation of the supernatant were observed.

Cumulative drug release from Cur in vitro was measured by dialysis. Briefly, SCCI (containing 1 mg of Cur, 2 mL) was added to dialysis bags with a molecular weight cutoff of 3500 Da. The dialysis bags were immersed in 500 mL PBS solution with 0.5% Tween at pH 5.0, 6.5, and 7.4. After incubation and shaking at 37 °C, aliquots of release medium (500 μL) were collected at 0.5, 1, 2, 4, 6, 8, 10, and 12 h, and equal amounts of fresh release medium were replaced. The concentration of Cur was determined by HPLC.

### In vitro photothermal imaging

For the photothermal imaging experiments, PBS, Cur, ICG (10 μg/mL), CCI, and SCCI with the same ICG equivalent concentration were added to 1.5 mL of centrifuge tubes and exposed to laser irradiation (808 nm, 0.75 W/cm^2^) for 5 min. The temperature variation was recorded by the FLIR C3-X photothermal camera (FLIR Systems, Estonia). Subsequently, the prepared SCCI (5, 10, 25, 50, and 100 μg/mL) solution was added to 1.5 mL of centrifuge tubes and irradiated for 5 min under the same conditions. Furthermore, the photothermal stability of the nanoparticles was verified by repeated "turn-off" laser irradiation and the temperature variation of each sample over time was recorded by the FLIR C3-X photothermal camera.

### In vitro cytotoxicity

The MTT assay was carried out to evaluate the cytotoxicity of Cur, ICG, CCI, and SCCI with NIR. Briefly, 4T1 cells were seeded in 96-well plates at a density of 5,000 per well and incubated in 200.0 µL of medium at 37 °C for 24 h. After removal of the medium, 200.0 µL of the medium containing different concentrations of Cur, ICG, CCI, and SCCI were added to the wells. After incubation for 4 h, After incubation for 4 h, the NIR group was irradiated with laser (808 nm, 0.75 W/cm^2^) in the dark for 3 min, and the cells were further incubated for 20 h. After the medium was removed, 20.0 µL of MTT solution (5 mg/mL) and 180 µL of complete medium were added to the wells and incubated for 4 h at 37 °C. The medium was then replaced with 150.0 µL dimethyl sulfoxide to solubilize the formazan crystals. Absorbance at 490 nm was measured by microplate reader (Thermo Fisher, USA).

Live/Dead cytotoxicity kit was used to further visualize the viability and survival rates of 4T1 cells. Briefly, 4T1 cells were seeded into 12-well plates at a density of 5 × 10^4^ per well and incubated overnight. After discarding the medium, the culture-medium containing Cur, CCI and SCCI were added to the plates and incubated for 6 h. After 4 h of co-incubation, cells in the NIR group were irradiated with laser (808 nm, 0.75 W/cm^2^) for 3 min and cultured for another 2 h. After removal of the culture medium, the cells were washed three times with PBS. 5 µL Calcein-AM solution (2 mM) and 15 µL propyl iodide solution (1.5 mM) was added to 5 mL 1 × Assay Buffer to obtain the staining working solution. Each well was added with 200 µL staining working solution and incubated at 37 °C for 15 min. The stained cells were observed by fluorescence microscopy.

### Cellular uptake of SCCI

To assess the uptake of nanoparticles in 4T1 cells, we replaced ICG with RhB. 4T1 cells were seeded into confocal dishes at a density of 4 × 10^4^ and cultured for 24 h. Cells were treated with free Cur, free RhB, Cur@CaCO_3_-RhB, and SA/Cur@CaCO_3_-RhB for 4 h. Subsequently, we washed the cells with PBS for 3 times, fixed the cells with 4% paraformaldehyde for 10 min, and then stained the nuclei with DAPI. Cellular uptake ability was assessed by confocal laser-scanning microscopy (CLSM, Leica Microsystems, Wetzlar, Germany).

Furthermore, to further quantify cellular uptake by flow cytometry, we replaced Cur with coumarin 6 (C6). 4T1 cells were seeded into 12-well plates (5 × 10^4^ cells/well) and cultured for 12 h. We treated the cells with free C6, C6@CaCO_3_-ICG, and SA/C6@CaCO_3_-ICG for 4 h. Cells were subsequently washed twice with PBS and collected. Cellular uptake of nanoparticles was quantified by flow cytometry.

### Biodistribution of SCCI in lysosomes

To detect the biodistribution of nanoparticles in lysosomes, 4T1 cells were seeded in 12-well plates at a density of 5 × 10^4^ per well and cultured in medium for 24 h. The cells were treated with medium containing SCCI for 1, 2, and 4 h. After washing the cells with PBS for 3 times, the lysosomes were stained with Lyso-Tracker Red probe. Lysosomes and Cur were visually characterized by fluorescence microscopy.

### In vitro ROS detection

DCFH-DA serves as a sensor for intracellular ROS detection. Briefly, 4T1 cells were seeded into 6-well plates at a density of 1 × 10^5^ per well and cultured for 24 h. After removing the medium, cells were then incubated with medium containing ICG, CCI, and SCCI for 4 h. Then, the NIR group was irradiated with a laser (808 nm, 0.75 W/cm^2^) for 3 min. The cells were incubated with DCFH-DA (10 μM) for 20 min. Finally, the ROS production was observed by fluorescence microscope.

Furthermore, 4T1 cells were seeded at a density of 5 × 10^3^ per well in 96-well plates and incubated in 200.0 µL of medium for 24 h. Cells were then incubated with medium containing ICG, CCI and SCCI for 4 h. After the incubation, the NIR group was irradiated with NIR laser (808 nm, 0.75 W/cm^2^) in the dark for 3 min. Cells were washed three times with PBS and then incubated with DCFH-DA (10 μM) for 20 min. Fluorescence intensity at 525 nm was detected with a microplate reader at 488 nm excitation wavelength.

Finally, 4T1 cells were seeded at a density of 5 × 10^4^ per well in 24-well plates and incubated in 500.0 µL of medium for 24 h. The cells were treated with the same method as described above and incubated with DCFH-DA (10 μM) for 20 min. The fluorescence intensity of each group was determined by flow cytometry.

### Detection of the MMP

4T1 cells were seeded in 6-well plates at a density of 1 × 10^5^ per well and cultured for 24 h in 2.0 mL of medium. They were then incubated with medium containing Cur, CCI and SCCI for 6 h. For the NIR group, the cells were irradiated with laser (808 nm, 0.75 W/cm^2^) for 3 min after co-incubation for 4 h and continued to culture for 2 h. Cells were then stained with JC-1 (5 μg/mL) for 30 min and washed 3 times with PBS. Finally, the variation of the MMP was observed by fluorescence microscopy.

### Ca^2+^ production by SCCI

First, 4T1 cells were seeded into 6-well plates at a density of 1 × 10^5^ per well and cultured for 24 h, then the previous medium was replaced with medium containing CCI and SCCI. For the NIR group, the cells were irradiated with NIR laser (808 nm, 0.75 W/cm^2^) for 3 min after incubation for 4 h, and continued incubation for 2 h. The cells were washed with PBS for three times and stained with Fluo-3 AM Ca^2+^ fluorescent probe. Finally, visualization of Ca^2+^ was characterized by fluorescence microscopy. Furthermore, the fluorescence intensity after staining was analyzed by flow cytometry. Briefly, 4T1 cells were seeded into a 12-well plate (5 × 10^4^ cells/well) and cultured for 12 h. The remaining steps were the same as those described above. Cells were collected after staining with Fluo-3 AM Ca^2+^ fluorescence probe and the intensity of fluorescence signal was analyzed by flow cytometry to determine the intracellular Ca^2+^ content.

### Detection of intracellular ATP content

To observe the variation of ATP levels in 4T1 cells, 4T1 cells were seeded at a density of 2 × 10^5^ per well in 6-well plates containing 2.0 mL of medium and cultured for 24 h. After discarding the medium, the medium containing Cur, Cur@CaCO_3_, CCI and SCCI was added into the wells and incubated for 6 h. After washing the cells with PBS for 3 times, the cells were treated with ATP extract solution and centrifuged at 10,000 g for 10 min. The supernatant was obtained to determine the content of ATP in cells according to the instructions of the ATP detection kit.

### In vivo imaging and biodistribution assays

Two groups of tumor-bearing mice were studied for in vivo imaging and biological distribution. First, free ICG and SCCI were injected into the tail vein. The biodistribution of nanoparticles in tumor-bearing mice was then recorded at 1, 2, 4, 8, and 12 h after drug administration using a small animal imaging system. The mice were subsequently sacrificed and in vitro images of major organs (heart, liver, spleen, lung, kidney) and tumors were obtained.

### In vivo photothermal imaging

Tumor-bearing mice were administered with saline, CCI and SCCI (2.3 mg/kg of ICG equivalent, 100 μL) through tail vein injection. After 8 h of injection, the tumor site was irradiated with laser (808 nm, 0.75 W/cm^2^) for 5 min, and temperature variation was recorded with a photothermal camera.

### In vivo antitumor efficiency

When the primary tumor volume reached about 100 mm^3^, the mice were divided into 6 groups (n = 5): (1) saline, (2) NIR, (3) Cur, (4) CCI, (5) SCCI, and (6) SCCI + L (10 mg/kg of Cur equivalent, 100 μL). At 8 h after administration, NIR and SCCI + L groups were irradiated with NIR laser (808 nm, 0.75 W/cm^2^) for 5 min. The tumor size was measured by digital caliper every 2 days for 2 weeks.

After the experiment, the mice were euthanized. The tumor was resected and fixed with 4% paraformaldehyde for histological analysis, including hematoxylin–eosin (H&E) staining, TdT-mediated dUTP nick-end labeling (TUNEL) and Ki67 staining. Major organs (liver and kidney) of mice were removed and fixed with 4% paraformaldehyde for H&E staining.

Finally, sera from each group of mice were collected and the hepatotoxicity of SCCI was assessed by measuring the serum levels of alanine aminotransferase (ALT) and aspartate aminotransferase (AST).

### Statistical analysis

All data were shown as mean ± standard deviation (SD). Statistical analysis was performed using GraphPad Prism 7 (GraphPad Software, USA). Statistical analysis was conducted with Student’s t-test, one-way analysis of variance and Dunnett’s multiple-comparison test. A P-value < 0.05 was considered to be statistically significant differences.

## Results

### Fabrication and characterization of SCCI

In this study, CCI nanoparticles were prepared by the one-pot gas diffusion process. Briefly, the CO_2_ generated during the decomposition of NH_4_CO_3_ diffused into the reaction solution to form CO_3_^2−^, which reacted with dissolved Ca^2+^ to form CaCO_3_ nanoparticles loaded with Cur and ICG. As a qualified goalkeeper, SA was wrapped around the CCI surface by Ca^2+^ crosslinking. Finally, we have successfully fabricated Ca^2+^ nano-regulator SCCI.

**Fig. 2:**
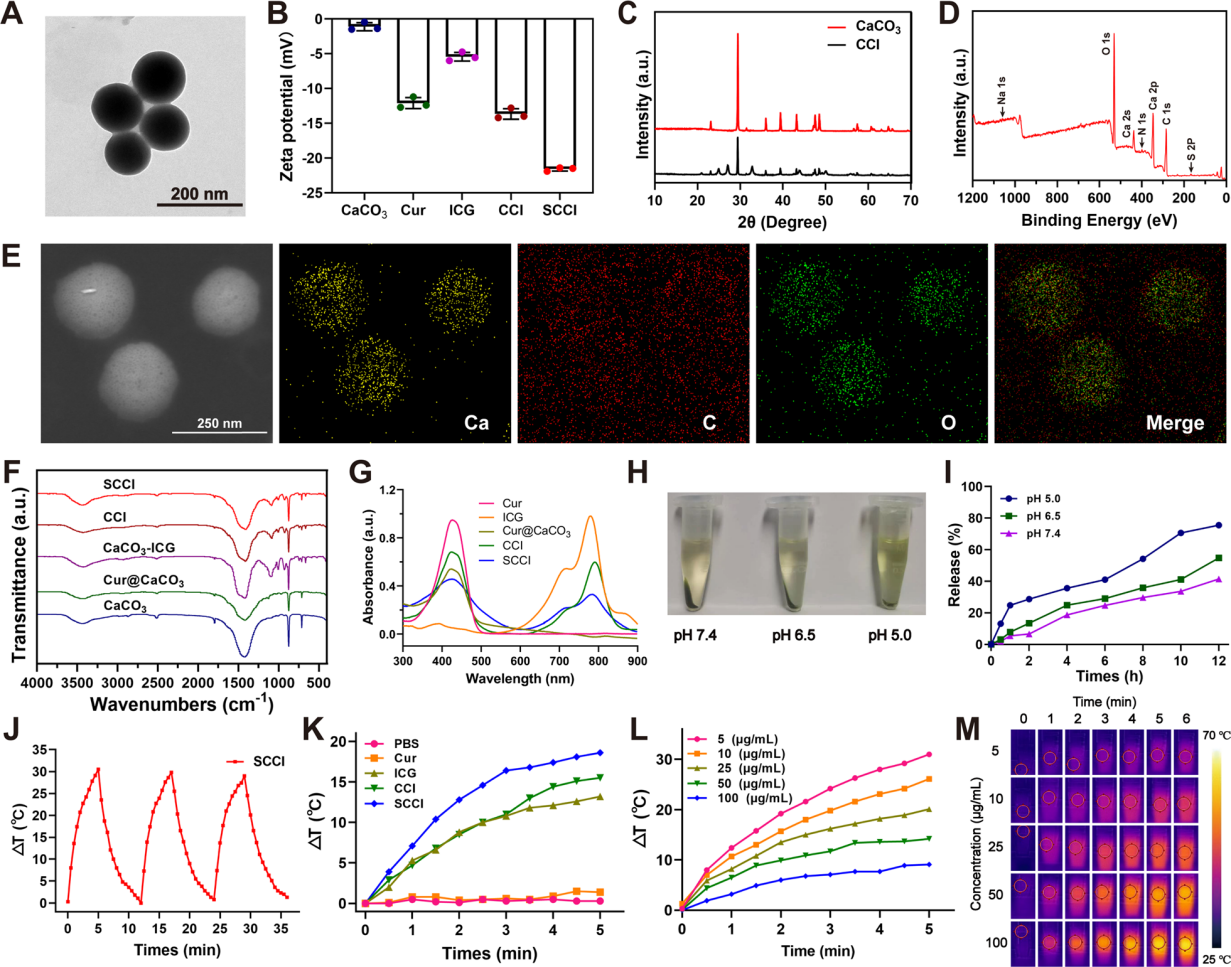
Preparation and characterization of SCCI. **A** TEM image of SCCI. Scale bar, 200 nm. **B** Zeta potential of CaCO_3_, Cur, ICG, CCI, SCCI. Data are shown as mean ± SD (n = 3). **C** XRD spectrum of CaCO_3_ and CCI. **D** XPS spectra of SCCI. **E** Elemental mapping of SCCI. Scale bar, 250 nm. **F** FT-IR spectra of CaCO_3_, Cur@CaCO_3_, CaCO_3_@ICG, CCI and SCCI. **G** UV–vis-NIR absorbance spectra for the Cur, ICG, Cur@CaCO_3_, CCI, and SCCI. **H** Pictures of SCCI dispersed in PBS solutions of pH 5.0, 6.5 and 7.4 for 1 h and centrifuged for 10 min. **I** Time-dependent release profiles of Cur in SCCI incubated in PBS at different pH values. Data are shown as mean ± SD (n = 3). **J** Heating curve of SCCI solution for three on–off cycles with an 808 nm NIR laser (0.75 W/cm^2^, 5 min). **K** Photothermal heating curves of PBS, Cur, ICG, CCI and SCCI under an 808 nm laser irradiation (0.75 W/cm^2^, 5 min). **L** Temperature curve of SCCI solution with different concentration under 808 nm NIR laser irradiation. (0.75 W/cm^2^, 5 min). **M** Thermographic images of different concentrations of SCCI under 808 nm NIR laser irradiation. (0.75 W/cm^2^, 5 min)

As shown in Fig. 2A and Additional file 1: Fig. S1, TEM results displayed that CCI and SCCI were monodisperse spherical nanoparticles with particle sizes of ~ 161 nm and ~ 175 nm, respectively. Moreover, it can be observed that SA uniformly covers the surface of CCI, enabling efficient encapsulation of Cur and ICG. The zeta potential results indicated that the addition of SA significantly increased the surface negative charge of CCI from − 13.7 mV to − 21.6 mV, which enhanced the stability of nanoparticles (Fig. 2B). XRD was used to investigate the structure of CaCO_3_. As exhibited in Fig. 2C, the diffraction peaks of CaCO_3_ appeared at 2θ of 23.10°, 29.45°, 36.02°, 39.46°, 43.21°, 47.53°, and 48.56°, which indicated that the prepared calcium carbonate crystal form was mainly calcite. Moreover, the analysis of CCI structure showed that the loading of Cur and ICG decreased the sharpness and intensity of the diffraction peaks of CaCO_3_, but did not change the crystal form of calcium carbonate in general. EDX analysis results indicated that SCCI was mainly composed of Ca, C, O and other elements, and these elements were uniform distributed in the structure of SCCI (Fig. 2E). The chemical composition of SCCI was further analyzed by XPS. The results were demonstrated in Fig. 2D and Additional file 1: Fig. S2, where the XPS spectra clearly suggested the presence of six elements: Ca, C, O, N, S, and Na. Among these elements, N and S originated from ICG and Na originated from SA, which fully proved the successful synthesis of SCCI.

**Fig. 3:**
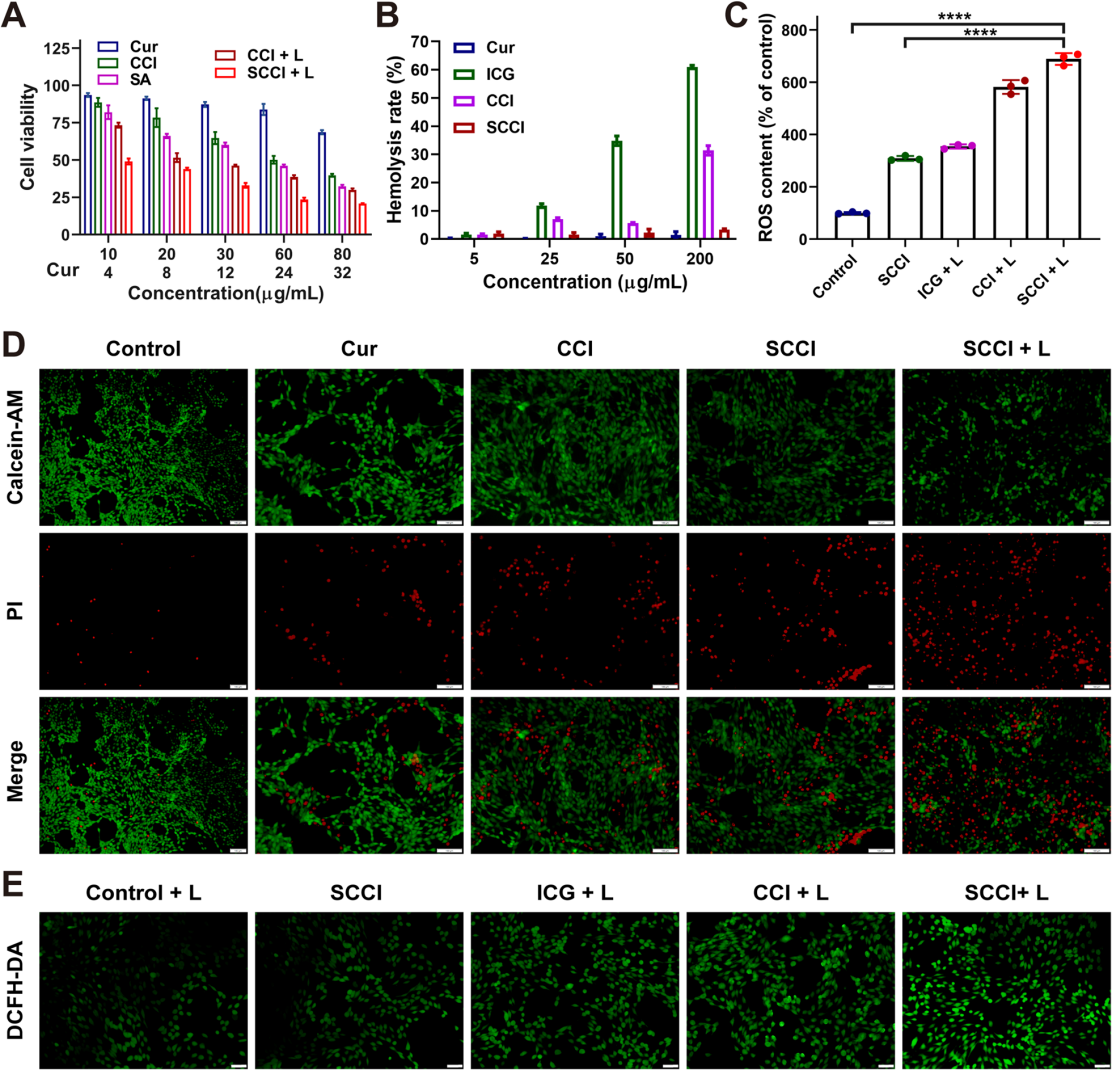
Ca^2+^ overload, PTT and ROS overproduction result in a synergistic enhancement of the cytotoxicity of SCCI nanoparticles. **A** Cytotoxicity of 4T1 cells treated with increasing concentrations of various formulations. Data are shown as mean ± SD (*n* = 3). **B** Hemolysis rate of different samples after incubation with RBC. **C** Fluorescence intensity analysis of DCFH-DA in different treated 4T1 cells with 808 nm laser irradiation. (0.75 W/cm^2^, 3 min). Data are shown as mean ± SD (*n* = 3). ****P < 0.0001. **D** Calcein-AM/PI live/dead double staining of 4T1 cells incubated with Cur, CCI, SCCI. Untreated cells served as the control group, and SCCI + L group was irradiated with an 808 nm laser after administration (0.75 W/cm^2^, 3 min). Scale bar, 100 μm. **E** Fluorescence images of DCFH-DA in 4T1 cells treated with different formulations after 808 nm laser irradiation (0.75 W/cm^2^, 3 min). Scale bar, 100 μm

FT-IR spectroscopy was used to verify the interaction between Ca^2+^ nano-regulator SCCI with Cur and ICG, and to determine the mechanisms of drug encapsulation and SA surface coating. The FT-IR spectra of Cur, ICG, CaCO_3_, Cur@CaCO_3_, CaCO_3_@ICG, CCI and SCCI were exhibited in Fig. 2F and Additional file 1: Fig. S3. After the encapsulation of the nanoparticles, most of the characteristic peaks of the material will be masked. As displayed in the figures, characteristic peaks at 1603 cm^−1^ and 1029 cm^−1^ (C=C bending on the aromatic rings) and 1429 cm^−1^ (C=C stretching in aromatic rings) derived from Cur appeared on the spectra of Cur@CaCO_3_ and CCI. In addition, the characteristic peaks at 1191 cm^−1^ (antisymmetric stretching vibration peak of sulfonic acid group) and 1089 cm^−1^ (symmetric stretching vibration peak of sulfonic acid group) derived from ICG appear on the spectra of CaCO_3_@ICG and CCI. These results confirmed that Cur and ICG were simultaneously loaded into CaCO_3_. Finally, the absorption peaks at 1623 cm^−1^ and 1413 cm^−1^ on the FT-IR spectrum of SCCI were antisymmetric stretching vibration peaks and symmetric stretching vibration peaks of SA carboxyl group, indicating that SA was successfully encapsulated on the surface of CCI. According to the results of HPLC, the LC of Cur was 40.7%, and the LE was 77.63%. Characteristic peak detection of SCCI using UV–vis absorption spectrum indicated that the characteristic peaks of Cur at 426 nm (Fig. 2G) [44]. In addition, the characteristic peak of ICG appeared at 780 nm in the near infrared region [45]. It is confirmed that the CaCO_3_ nanoparticles achieve simultaneous loading of Cur and ICG. The LE of ICG as determined by UV–vis absorption spectroscopy was 78.04% (Additional file 1: Fig. S4). Hemolysis assay was used to assess the biocompatibility of nanomaterials in the range of 5 to 200 μg/mL. As shown in Fig. 3B, the positive control group was completely hemolytic as compared with the negative control group. The free ICG exhibited a high rate of hemolysis, with more RBC rupturing as its concentration increased. Moreover, RBC hemolysis rate of SCCI was low, even at 200 μg/mL the hemolysis rate was less than 5% (Additional file 1: Fig. S5). These results indicated that the encapsulation of CaCO_3_ nanoparticles by SA significantly reduces the hemolysis rates of ICG and CCI. The SCCI prepared in this study is well biocompatible.

### Release of Ca^2+^ and Cur in acidic environments

As a pH-responsive release carrier, SCCI was able to decompose and release Ca^2+^ and Cur in the acidic environment of tumors. In hence, we investigated the decomposition of SCCI placed at pH 5.0, 6.5, and 7.4 for 1 h. As displayed in Fig. 2H, after centrifugation of the sample, the color of the supernatant of the solution in the centrifuge tube deepened and precipitation decreased with the decrease of the solution pH. Furthermore, to validate the pH-sensitive release properties of Cur in SCCI, we investigated the cumulative drug release of Cur in vitro by immersing SCCI in solutions of pH 5.0, 6.5, and 7.4. In Fig. 2I, the cumulative release of Cur was around 40% after 12 h of placing SCCI into a solution of pH 7.4, indicating the efficient encapsulation of the nanoparticles by SA. In contrast, in pH 5.0 and pH 6.5 solutions, the Cur cumulative releases at different time points were significantly higher than those at pH 7.4, and the final cumulative releases exceeded 70% and 50%, respectively. These results suggested that Ca^2+^ nano-modulators, based on their pH-responsive decomposition properties, accelerate disintegration and release more Ca^2+^ and Cur in the acidic microenvironment of tumor cells, overloading Ca^2+^ in mitochondria.

### Photothermal conversion activity of SCCI

Thermal production efficiency is an important index to evaluate the photothermal efficiency [46]. The strong absorption of the 808 nm laser by ICG enables the conversion of optical energy to heat energy, which damages cells by heating up sharply. To verify whether the ICG embedded in the CaCO_3_ retains its original optical properties. We need to evaluate the photothermal conversion performance of SCCI. The temperature variation during the test period were recorded by the FLIR photothermal camera. Figure 2K suggested that only the samples containing ICG showed significant warming upon irradiation with the NIR (0.75 W/cm^2^). In addition, after 5 min of NIR irradiation, the temperature of SCCI increased by 18.6 °C, significantly higher than the 13.2 °C of ICG, revealing a better photothermal conversion capacity. Further studies demonstrated (Fig. 2L and M) that the thermogenic level of SCCI increased with increasing concentration, showing obvious concentration dependence. After exposure to 0.75 W/cm^2^ NIR for 5 min, the temperature of SCCI at 50 μg/mL increased by 26.1 °C, significantly exceeding the high temperature that breast cancer cells can withstand [47]. As exhibited in Fig. 2F, SCCI had superior optical stability. The temperature rise of nanoparticles did not change significantly under three consecutive on–off laser cycles. The above results demonstrated that the method of embedding the ICG into CaCO_3_ and efficiently encapsulating it with SA not only preserved the optical properties of the ICG, but also further enhanced the photostability, creating an ideal agent for photothermal therapy. After being ingested by breast cancer cells, the nanoparticles can directly kill breast cancer cells after sustained NIR treatment.

### Cytotoxicity evaluations of SCCI

Based on the mitochondrial Ca^2+^ overload and significant photothermal effects, the SCCI demonstrated a robust tumor suppression capacity. To assess the cytotoxicity of SCCI, we performed a comprehensive cytotoxicity assessment of the preparations at different concentrations by MTT assay. The results revealed that the cytotoxicity of CCI and SCCI were dose-dependent with increasing Cur and Ca^2+^ concentrations (Fig. 3A). Compared with Cur at the same concentration, SCCI exhibited more cytotoxicity. Furthermore, external laser irradiation enhanced cytotoxicity by warming up and promoting Ca^2+^production. Even at a lower concentration (30 μg/mL), 4T1 cells were effectively killed by NIR laser irradiation. When the concentration of Cur varied from 5.6 to 22.4 μg/mL, the cytotoxicity of Cur, SCCI and SCCL + L groups were extremely significant differences, indicating that the synergistic effect of chemotherapy, photothermal therapy and mitochondrial Ca^2+^ overload could effectively kill 4T1 cells. In addition, live/dead cell staining assay also suggested that SCCI nanoparticles had superior PTT effect (Fig. 3D). In this experiment, living cells were dyed green and dead cells red. Compared with the control group, SCCI without laser irradiation exhibited significant cytotoxicity. However, the laser-induced PTT effect was able to kill more 4T1 cells, which coincided with the MTT results. The results of the aforementioned cytotoxicity studies indicated that SCCI killed most of the tumor cells through Ca^2+^ overload and photothermal effect, causing damage to the tumor tissue.

### Detection and analysis of ROS in vitro

DCFH-DA was used as a ROS sensor and intracellular ROS generation was detected by fluorescence microscopy [48]. As displayed in Fig. 3E, the untreated control group only emitted weak green fluorescence. Nanomaterials containing ICG, on the other hand, produced bright green fluorescence under laser irradiation, which represented more ROS production. In addition, CCI and SCCI produced more ROS than ICG, since the stability of free ICGs was lower, and the effective coating of SA enhanced the stability of ICG in CaCO_3_. This was further confirmed in the quantitative analysis of microplate reader in Fig. 3C, where the SCCI + L group generated approximately 6.8 times more ROS than the control group and 1.9 times more than ICG + L. The results of flow cytometry showed the greatest fluorescence intensity in the SCCI + L group, indicating the production of massive ROS (Additional file 1: Fig. S6). The fluorescence intensity in this group was 5.8 times that of the control group and 2.5 times that of the ICG + L group, in agreement with the microplate reader results. The above studies indicated that SCCI was able to produce large amounts of ROS under the irradiation of the NIR laser, further aggravating the cytotoxicity.

### Generation of Ca^2+^ from SCCI in cells

**Fig. 4:**
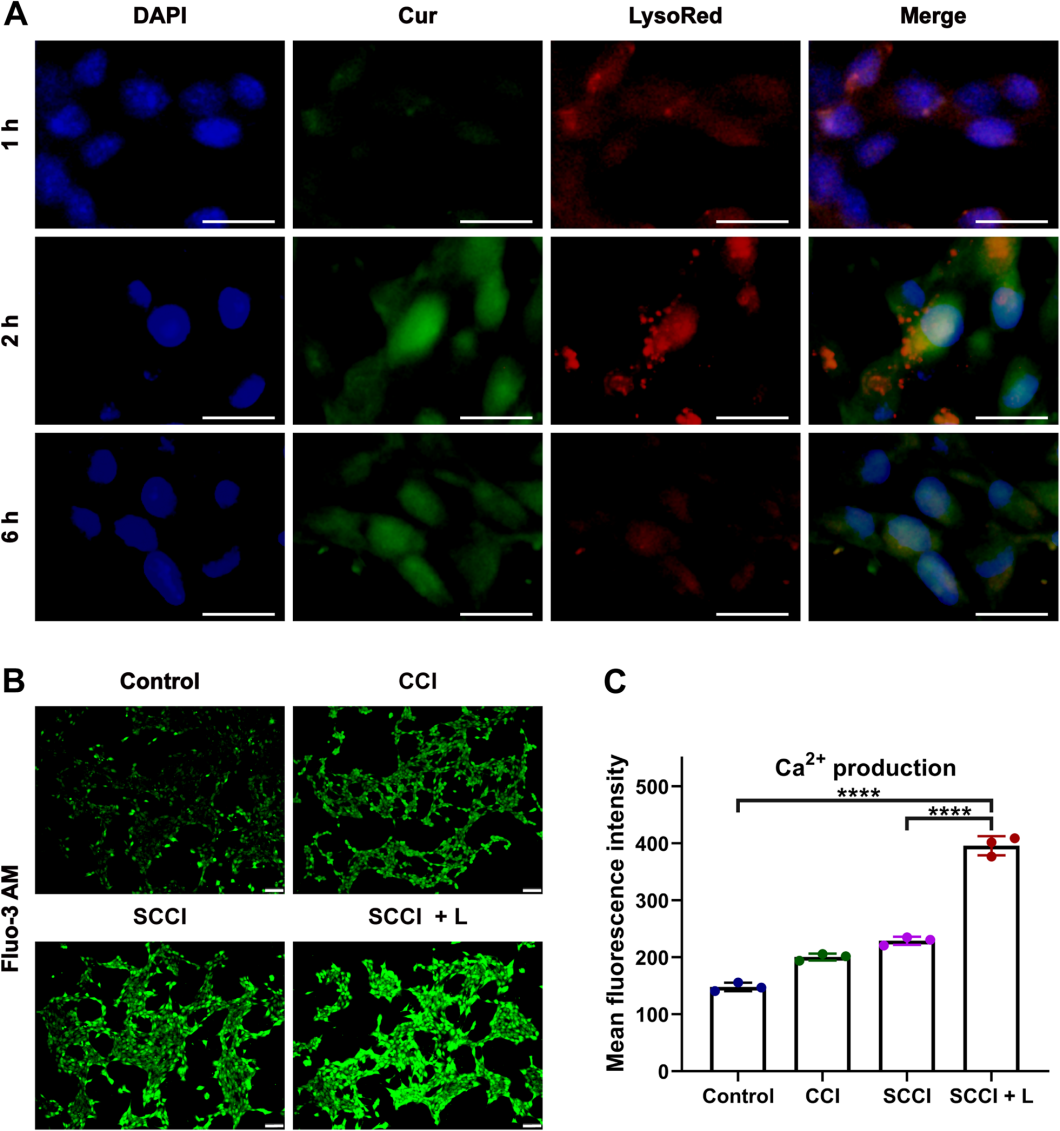
SCCI releases CUR and Ca^2+^ by consuming H^+^ in lysosomes. **A** SCCI in lysosomes for different time treatment to 4T1 cells. Scale bar: 20 μm. **B** Fluorescence microscope images of intracellular Ca^2+^ production after 6 h treatment with CCI and SCCI. SCCI + L group was irradiated with an 808 nm laser after administration (0.75 W/cm^2^, 3 min). Scale bar, 100 μm. **C** The mean fluorescence intensity of intracellular Ca^2+^ production quantified by flow cytometry. Data are shown as mean ± SD (*n* = 3). ****P < 0.0001

To determine the distribution of SCCI in lysosomes and the generation of Ca^2+^, we used Lyso-Tracker Red probes with red fluorescence to specifically label lysosomes and DAPI with blue fluorescence to stain the nuclei, and observed the intracellular behavior of the Cur with green fluorescence [49]. As displayed in Fig. 4A, the colocation rate of SCCI with lysosomes increased with time and was highest at 4 h. However, after 6 h the fluorescence of lysosomes and Cur gradually separated, the colocation rate decreased significantly and only a small part of Cur entered into the nucleus. The results revealed that Ca^2+^ nano-regulator SCCI continuously depleted H^+^ and released Ca^2+^ and Cur in the acidic environment of lysosomes, achieving escape from lysosomes.

Based on the above findings, we speculated that SCCI released large amounts of Ca^2+^ and Cur, coordinating the promotion of mitochondrial Ca^2+^ overload. In consequence, we tracked the intracellular Ca^2+^ generation by using Fluo-3 AM fluorescent probe. Fluo-3 AM is able to be cleaved by esterase to form Fluo-3, which produces strong fluorescence after binding with intracellular Ca^2+^. As exhibited in Additional file 1: Fig. S7, the fluorescence intensity of the Cur group increased compared to the control group, resulting from the release of Ca^2+^ from the ER and the efflux of Ca^2+^. Due to the synergistic effect of Cur and CaCO_3_, the fluorescence intensity in the CCI group was significantly higher than other groups. In Fig. 4B, the fluorescence intensity of the SCCI group was higher than that of the CCI group, and under the irradiation of 808 nm laser, the SCCL + L group had the strongest fluorescence intensity. Subsequently, the fluorescence intensity of each group was then quantified by flow cytometry. As demonstrated in Fig. 4C, the fluorescence intensity of Ca^2+^ after SCCI treatment was 229. When NIR irradiation was administered for 3 min, the fluorescence intensity of intracellular Ca^2+^ increased sharply to 396. The results confirmed that the uptake of SCCI by cells could release large amounts of Ca^2+^, and NIR laser could accelerate the decomposition of CaCO_3_, which was conducive to play the synergistic role of Cur.

### Breakdown of mitochondrial Ca^2+^ homeostasis SCCI

**Fig. 5:**
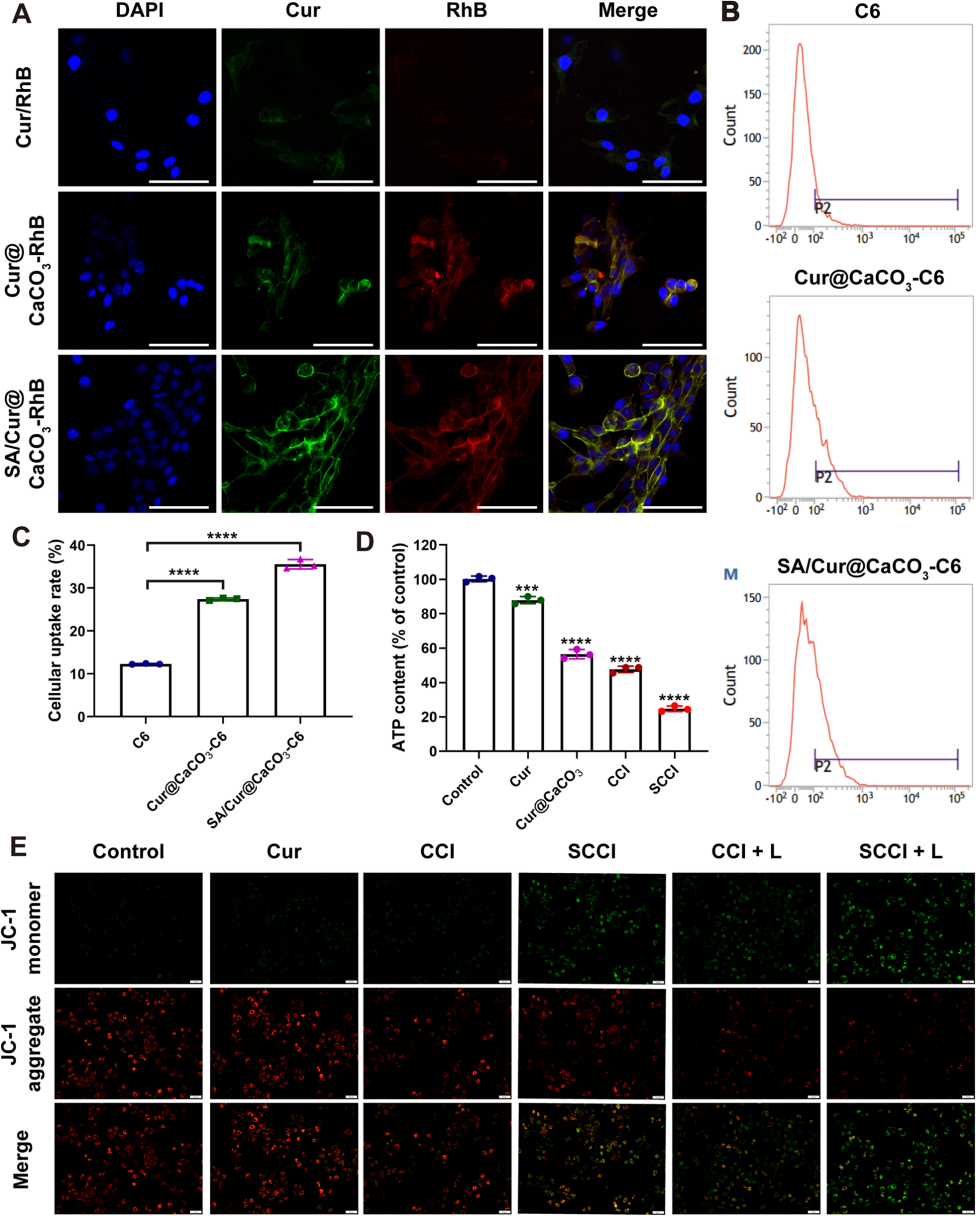
In vitro intracellular uptake and MMP detection. **A** CLSM of the uptake of Cur@CaCO_3_-RhB and SA/Cur@CaCO_3_-RhB by 4T1 cells. Scale bar, 75 μm. **B** Cellular uptake of C6, Cur@CaCO_3_-C6 and SA/Cur@CaCO_3_-C6 quantified by flow cytometry. **C** Analysis of the cellular uptake rate of C6, Cur@CaCO_3_-C6 and SA/Cur@CaCO_3_-C6 in 4T1 cells. Data are shown as mean ± SD (*n* = 3). ****P < 0.0001. **D** Analysis of intracellular ATP content after treatment with different formulations. Data are shown as mean ± SD (*n* = 3). ***P < 0.001, ****P < 0.0001. **E** MMP detection of 4T1 cells treated with different preparations. CCI + L group and SCCI + L group were irradiated by 808 nm laser after administration. Scale bar, 50 μm

In order to better evaluate the cellular uptake of SCCI by 4T1 tumor cells, we selected RhB to replace ICG. 4T1 cells were treated with free Cur, RhB, Cur@CaCO_3_-RhB and SA/Cur@CaCO_3_-RhB for 4 h. The distribution of Cur and RhB in cells were observed by CLSM, and fluorescence quantitative analysis was performed by flow cytometry. As exhibited in Fig. 5A, the fluorescence intensity of Cur (green fluorescence) and RhB (red fluorescence) in the SA/Cur@CaCO_3_-RhB group was significantly higher than the other two groups, while the free Cur and RhB showed only weak fluorescence. The results of flow cytometry also confirmed this view (Fig. 5B and C). These results indicated that SCCI can be efficiently internalized by 4T1 cells and subsequently exert its therapeutic effect.

To further explore the apoptosis mechanism by which SCCI disrupted mitochondrial homeostasis, we used the JC-1 MMP detection kit to detect the effect of Ca^2+^ and laser irradiation on the mitochondria of 4T1 cells. When the MMP is high, JC-1 is able to accumulate in the mitochondrial matrix to form a polymer that produces red fluorescence. Conversely, at a lower membrane potential, JC-1 exists as a monomer and produces green fluorescence [50]. As displayed in Fig. 5E, the green fluorescence intensity was increased in the 4T1 cells when treated with Cur alone. When treated with SCCI, the cells exhibited a stronger green fluorescence with the combination of CaCO_3_ and Cur. In addition, under laser irradiation, the JC-1 monomer/aggregation ratio increased significantly in both CCI and SCCI groups, with the highest JC-1 monomer/aggregation ratio in the SCCI group, resulting in severe mitochondrial damage. These results indicated that the combined effects of Cur, CaCO_3_ and NIR significantly reduced MMP, disrupted mitochondrial homeostasis and induced its apoptosis.

Life activity and proliferation in tumor cells require substantial ATP support. And the structural damage and dysfunction of mitochondria inevitably lead to a sharp decrease in ATP content [51]. Consequently, we also investigated the cellular ATP levels after treatment with different formulations by ATP assay kit. The results were exhibited in Fig. 5D, with the exception of the Cur group, where the ATP content in 4T1 cells decreased differently compared to the control group. Especially in the SCCI group, the relative ATP content decreased the most, by only 24.7% of that in the control group. These data suggested that Ca^2+^ nano-regulators significantly disrupted mitochondrial homeostasis, exacerbated cell damage, and sharply cut off energy supply to tumor cells.

### In vivo imaging and biodistribution studies

**Fig. 6:**
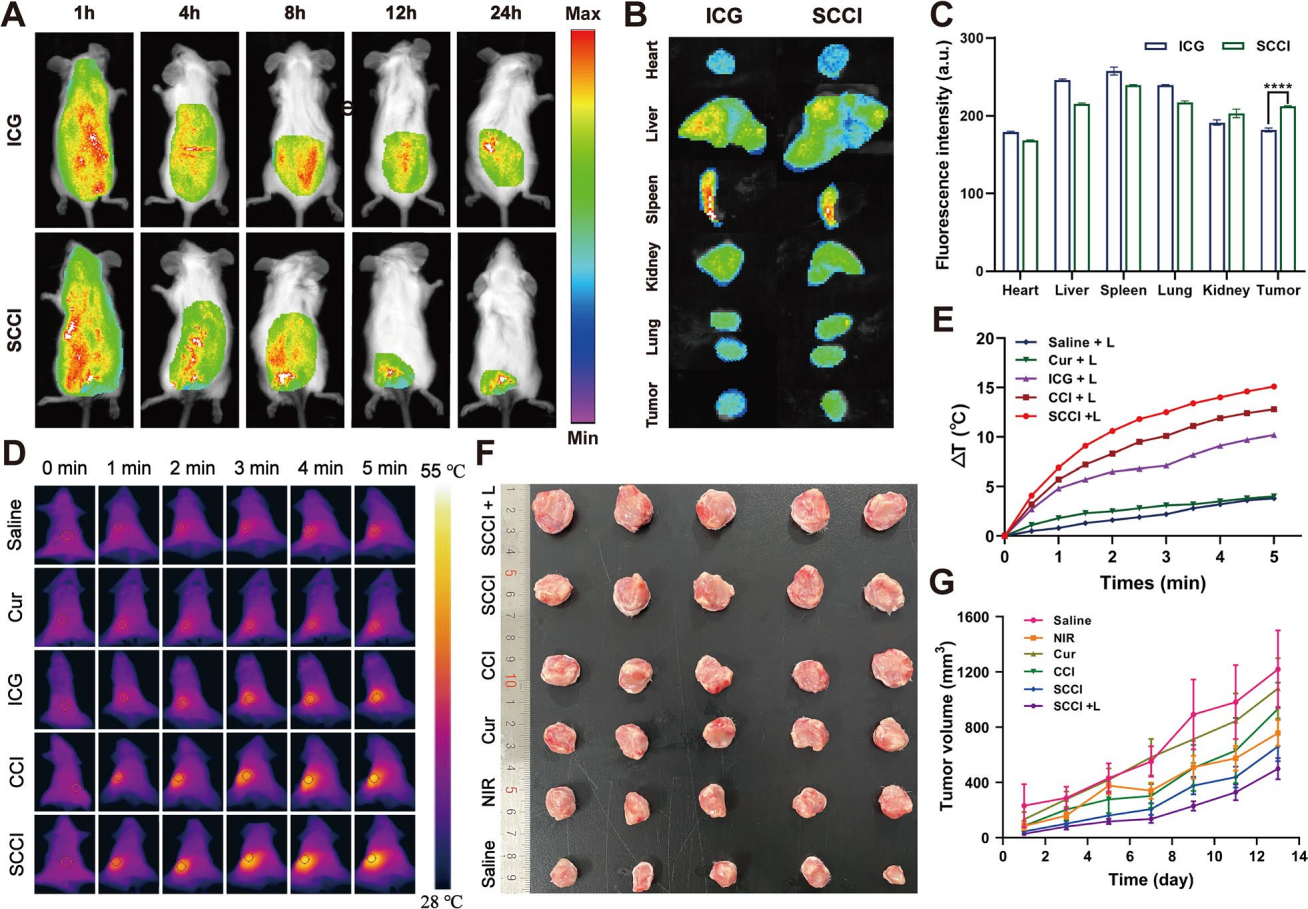
In vivo synergistic therapy of SCCI combined with NIR irradiation. **A** In vivo fluorescence images of tumor-bearing mice after intravenous injection of ICG and SCCI at the indicated time points. **B** Ex vivo fluorescence images of major organs (heart, liver, spleen, lung, kidney) and tumors after ICG and SCCI at 24 h post-injection. **C** Fluorescence intensity of isolated organs and tumors was quantified 24 h after injection. Data are shown as mean ± SD (*n* = 3). ****P < 0.0001. **D** Photothermal images of tumor sites after injected with different formulations (0.75W/cm^2^, 5 min). **E** Photothermal heating curves of tumor-bearing mice after injected with saline, Cur, ICG, CCI and SCCI with 808 nm laser irradiation (0.75W/cm^2^, 5 min). **F** In vitro tumor images of each group 24 h after the last administration. Data are shown as mean ± SD (n = 5). **G** Tumor volume variation in 4T1 tumor-bearing mice after treatment with different formulations. Data are shown as mean ± SD (n = 5)

The effective accumulation of Ca^2+^ nano-modulators at the tumor site is particularly important for the efficacy of the treatment. To this end, we tracked the biological distribution of SCCI by in vivo fluorescence imaging. As exhibited in Fig. 6A, the fluorescence intensity was significantly higher in the SCCI group than in the ICG group at all time points. This indicated that SCCI nanoparticles can maintain sustained fluorescence signal due to long-term circulation and efficient accumulation in tumor tissue. In addition, to achieve the desired thermal ablation effect of the tumor cells, it is necessary to determine the appropriate duration of the NIR laser irradiation. We found that the intensity of the fluorescence signal was significantly higher in the SCCI group than at other time points 8 h after administration. Therefore, in the following in vivo photothermal imaging and anti-tumor activity studies, we chose to irradiate the tumor site with NIR laser 8 h after administration. At 24 h after the administration, the mice were sacrificed, and the main organs (heart, liver, spleen, lung and kidney) and tumors were harvested for ex vivo fluorescence imaging. The results were displayed in Fig. 6B and C, the fluorescence intensity of SCCI in tumor tissue was significantly increased compared with the ICG, which further proved the superior enrichment ability of SCCI at tumor sites. These results suggested that the high distribution and long circulation of SCCI in tumors gave it a great advantage in the delivery of antitumor drugs.

### In vivo photothermal effect of SCCI

To assess the photothermal effect of SCCI at the tumor site, we captured thermal imaging maps of the tumor site using a formal camera and recorded the temperature variation. As demonstrated in Fig. 6D and E, the tumor sites of mice in saline and Cur groups, respectively, increased by 3.8 °C and 4 °C, within 5 min under NIR irradiation (0.75 W/cm^2^). The temperature of the tumor site in the nanomaterial group containing ICG increased significantly, among which the CCI and SCCI groups increased by 12.8 °C and 15.1 °C, respectively. Studies have suggested that the PTT effect based on photothermal transformation nanomaterials can directly combat tumor cells through thermal ablation at temperatures of 40 ~ 47 °C [52, 53]. In consequence, only by controlling the appropriate temperature and time, SCCI based photothermal therapy was able to effectively kill tumor cells without damaging normal cells and tissues.

### Antitumor effects of SCCI in vivo

To further explore the antitumor effects of SCCI in mice, we established a 4T1 orthotopic breast cancer model in BALB/c mice. Briefly, tumor-bearing mice were randomized into the following 6 groups and given (1) saline, (2) NIR, (3) Cur, (4) CCI, (5) SCCI, and (6) SCCI + L. As shown in Additional file 1: Fig. S8, the first dose was initiated when the primary tumor volume reached approximately 100 mm^3^, and was then administered every other day for 2 weeks. The body weight of tumor-bearing mice during treatment changed over time as displayed in Additional file 1: Fig. S9. All mice demonstrated an increase in body weight during treatment, indicating that the constructed Ca^2+^ nano-regulators were less toxic and had excellent biocompatibility. As shown in Fig. 6F and Additional file 1: Fig. S10, the treatment effect of Cur was less pronounced and the tumor volume was slightly reduced compared to the control group. However, the NIR group and the CCI group exhibited some therapeutic inhibition effects, with a tumor volume of 757.76 ± 83.10 mm^3^ and 933.74 ± 169.19 mm^3^, respectively (Fig. 6G). SCCI has been able to exert therapeutic effects through photothermal conversion under NIR irradiation. Consequently, compared with the SCCI, the SCCI + L had a more powerful tumor inhibition effect, and the tumor volume was 499.64 ± 69.18 mm^3^. The weight distribution of isolated tumors also suggested that SCCI and SCCI + L groups had strong inhibitory effect on tumor growth, which was 0.9084 ± 0.1002 g and 0.5146 ± 0.1840 g, respectively (Additional file 1: Fig. S11). These results indicate that Ca^2+^ nano-modulators can effectively kill tumor cells in combination with chemotherapy, PTT, and mitochondrial Ca^2+^ overload, in agreement with cell experiments.

**Fig. 7:**
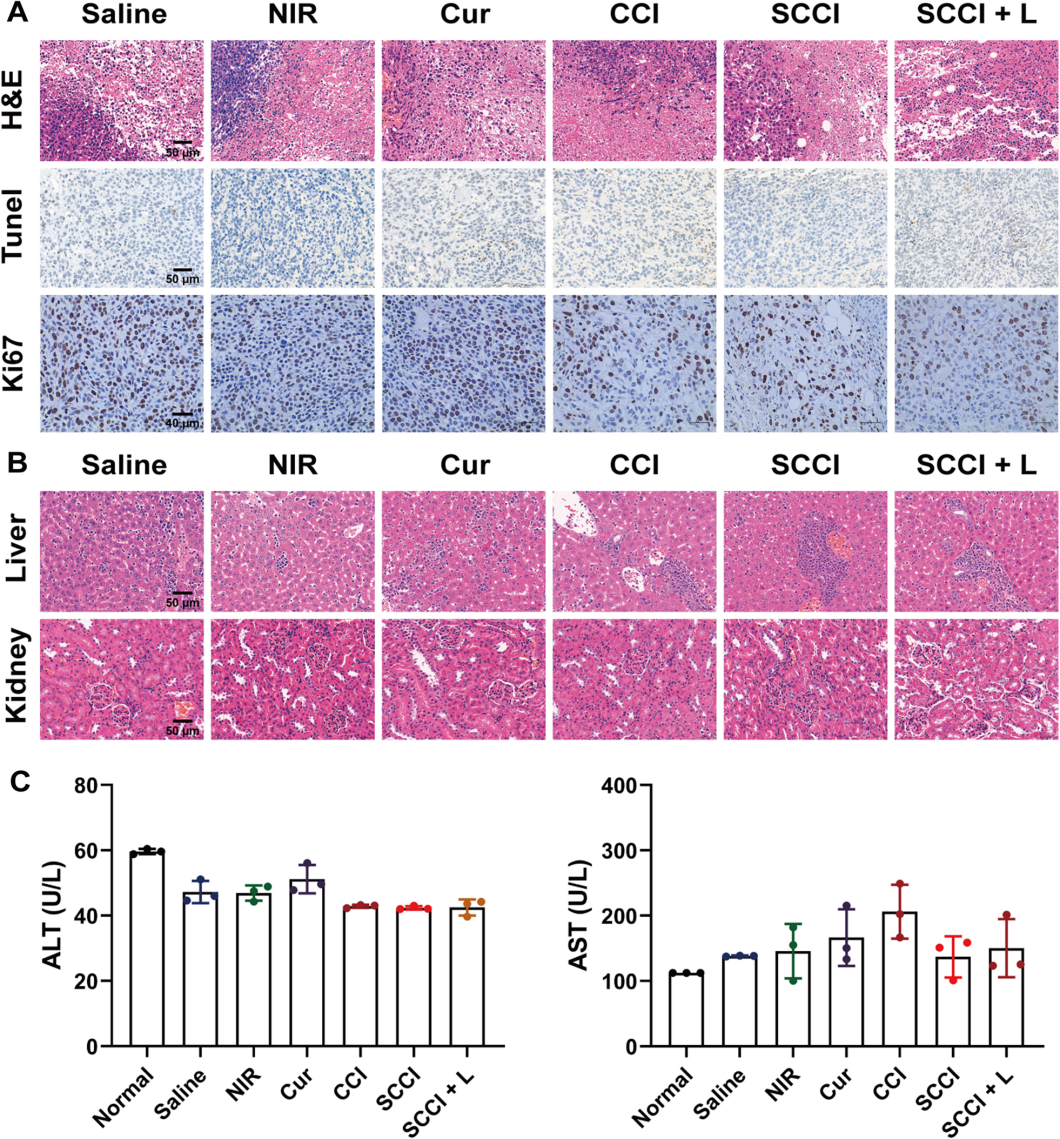
Histological analysis and safety evaluation. **A** Representative H&E, TUNEL and Ki67 staining images of exfoliated tumor tissue in different treatment groups. Scale bars, 50 μm for H&E and TUNEL images and 40 μm for Ki67 images. **B** Representative H&E staining images of exfoliated hearts and livers in different treatment groups. Scale bar, 50 μm. **C** Serum levels of AST and ALT in tumor-bearing mice with different treatment. Data are shown as mean ± SD (n = 3)

We further investigated the antitumor effect of SCCI by pathologic analysis of the tumors by H&E and TUNEL staining (Fig. 7A). In the normal saline group, the tumor tissues were closely arranged, the morphology were complete, and the pathological features were apparent. In contrast, different degrees of nuclear fissure and necrosis appeared in the tumor tissues of the other treatment groups, with blurred tumor cell boundaries and reduced cell number. As expected, the tumor tissues in the SCCI + L group were the most severely damaged, with intertissue fluid and space in the cytoplasm, and massive cell apoptosis. Ki67 immunohistochemical staining demonstrated that proliferation of 4T1 cells was significantly inhibited in the SCCI and SCCI + L groups, and the percentage of Ki67 positive cells in the SCCI and SCCI + L groups was significantly lower than in the normal saline group (Additional file 1: Fig. S12). These results confirmed the powerful antitumor effect of Ca^2+^ nano-regulator SCCI, in combined with chemotherapy, PTT and mitochondrial Ca^2+^ overload.

To assess the potential toxicity of Ca^2+^ nano-modulators in vivo, we stained major organs using H&E. As shown in Fig. 7B and Additional file 1: Fig. S13, the livers and kidneys of the saline group and each treatment group demonstrated no significant pathological abnormalities and lesions, indicating good biocompatibility. The measured values of serum AST and ALT in SCCI and SCCI + L groups were within the normal range and exhibited no significant difference between normal mice, further confirming the safety of SCCI (Fig. 7C).

## Discussion

In this study, we successfully loaded Cur and ICG into CaCO_3_ through the one-pot gas diffusion process. By wrapping the CaCO_3_ with SA via Ca^2+^ crosslinking, SCCI was successfully constructed. This Ca^2+^ nano-modulator SCCI integrates chemotherapy, PTT and mitochondrial Ca^2+^ overload to coordinate anti-tumor effects, demonstrating strong anti-tumor capabilities.

SCCI exhibited a stable homogeneous spherical structure as observed by the results of TEM images. The results of EDX and XPS analyses exhibited that Ca, C and O elements were uniformly distributed in the SCCI structure, which constituted the bulk of the nanoparticles. The existence of other elements confirmed the successful synthesis of SCCI. Both Cur and ICG were efficiently loaded into CaCO_3_ nanoparticles with loading efficiencies of 77.63% and 78.04%, respectively, as indicated by UV–vis absorption spectroscopy and FT-IR detection. This high loading amount can be attributed to the porous nanostructure of CaCO_3_. In vitro and in vivo photothermal conversion studies revealed that, benefiting from the high loading amount and the coating of SA, SCCI not only maximized the photothermal performance of ICG and enhances its stability, but also promoted the faster decomposition of nanoparticles under the irradiation of NIR and accelerates the process of mitochondrial Ca^2+^ overload. Compared to free ICG and CCI, SCCI can not only play a stronger role in directly killing tumor cells under NIR laser irradiation, but also induce tumor cell apoptosis by generating additional ROS. Moreover, this high loading amount was also beneficial for enhancing tumor cell cytotoxicity. Results from MMT and Live/Dead cytotoxicity assays indicated that SCCI + L killed more than 50% of 4T1 cells even at the lowest concentration, which was attributed to strong PTT effects and Ca^2+^ overload. In contrast, SCCI exhibited excellent biocompatibility and biodegradability in blood circulation and major organs. Hemolysis assay suggested that the coating of SA significantly reduced the hemolysis rate of ICG and kept it within the maximum allowable limit. Moreover, there were no significant pathological abnormalities and lesions in the livers and kidneys in all groups.

Due to the acidic tumor microenvironment, pH response becomes one of the common trigger conditions [54–57]. Therefore, to investigate the pH-responsive release properties of CaCO_3_ nanoparticles, we simulated the environment in tumor and cellular lysosomes with acidic pH and examined the in vitro release behavior of Ca^2+^ nano-modulator SCCI at pH 5.0, 6.5, and 7.4. The rate of Ca^2+^ and Cur release increased with decreasing pH, which facilitated the absorption of drugs at weakly acidic tumor sites. Moreover, lysosomal biodistribution assay also demonstrated that SCCI continuously depleted H^+^ in the acidic environment of the lysosome and released Ca^2+^ and Cur, enabling lysosome escape. Cellular uptake and in vivo biodistribution assays demonstrated that coating of SA allowed SCCI to be more efficiently internalized by 4T1 cells, with a broader distribution and stronger binding capacity in tumor tissue. After being effectively internalized into the tumor, SCCI can significantly reduce MMP, disrupt mitochondrial homeostasis, and cut off the energy supply by acting as a co-ordinator between PTT and mitochondrial Ca^2+^ overload. In in vivo antitumor studies, SCCI has demonstrated strong inhibition and killing ability of tumor cells. Based on the excellent photothermal conversion efficiency of ICG, the tumor site was significantly heated under the irradiation of NIR laser, which enhanced the tumor suppression through thermal ablation. Tumor growth in the SCCI + L group was significantly inhibited compared to the saline group, which also confirmed the favorable tumor suppression effect of the Ca^2+^ nano-regulators. H&E and TUNEL staining results demonstrated that the tumors in SCCI + L group were the most severely damaged, with large amounts of cells showing nucleation and spotty necrosis. Ki67 immunohistochemical staining also confirmed a significant inhibition of tumor cell proliferation in the SCCI + L group.

Recently, several studies have been reported on the coordinated role of Cur and calcium-containing nanocarriers in promoting mitochondrial Ca^2+^ overload [58–60]. For example, Yin et al. loaded Cur and transferrin into calcium peroxide nanoparticles to achieve the overload therapy of Ca^2+^ [61]. By loading Cur with CaCO_3_ and MnO_2_ into nanoparticles coated with a cancer cell membrane, Luo et al. prepared a pH-responsive nano-modulator [62]. On the basis of previous studies, we loaded ICG and Cur into CaCO_3_ nanoparticles, and firstly linked PTT with a synergistic mitochondrial Ca^2+^ overload strategy to enhance antitumor effects. The photothermal conversion capability of ICG accelerated the decomposition of CaCO_3_, prompting a sharp increase of Ca^2+^ in mitochondria in a short time. By severely disrupting mitochondrial homeostasis and PTT, SCCI can simultaneously induce apoptosis and directly kill tumor cells. Moreover, the Ca^2+^ nano-regulators have obvious advantages in large-scale production, which is attributed to their abundant and easy availability of processed raw materials, simple preparation process and high yield. The coating of SA is capable of prolonging the circulating time of Ca^2+^ nano-regulators, increasing their accumulation at tumor sites, and improving their biocompatibility. However, relying solely on the EPR effect in tumor blood vessels, the current targeting of Ca^2+^ nano-regulators are insufficient to fully unleash their potential. Further improvements in the active targeting capability of the nanoparticles are required. Moreover, although more specific single-cell level investigations are needed to elaborate on the mechanisms of Ca^2+^ nano-regulators, this study provides considerable reference for the development of safe and effective antitumor nanoparticles.

## Conclusion

In summary, this study successfully constructed a multimodal Ca^2+^ nano-regulator SCCI with a large intracellular Ca^2+^ production capacity. The synthesized SCCI had a stable and homogeneous spherical structure with an average particle size of less than 200 nm. In vitro release experiments have suggested that SCCI had pH response decomposition properties. The acidic microenvironment of tumors can accelerate the decomposition of these nanoparticles and release large amounts of Ca^2+^ and Cur, synergistically aggravating the Ca^2+^ overload within mitochondria and inducing apoptosis of tumor cells. SCCI embedded with ICG showed a favorable photothermal effect. Under NIR irradiation, it is possible to not only accelerate the disintegration of nanoparticles and promote the generation of large amounts of ROS, but also directly kill tumor cells through substantial heating. In vivo antitumor results have suggested that the Ca^2+^ nano-modulators had high biosafety and strong tumor suppression and lethality. In conclusion, we believed that this precisely designed Ca^2+^ nano-regulators based on Ca^2+^ overload and PTT had great potential for mitochondrial targeted cancer therapy.

### Supplementary Information

**Additional file 1.** Additional figures.

## Data Availability

All data needed to support the conclusions are present in the paper or the Additional materials.

## Abbreviations

Cur: Curcumin
ER: Endoplasmic reticulum
ICG: Indocyanine green
CCI: Cur@CaCO_3_-ICG
SCCI: SA/Cur@CaCO_3_-ICG
SA: Sodium alginate
CaCO_3_: Calcium carbonate
NIR: Near-infrared light radiation
ROS: Reactive oxygen species
MMP: Mitochondrial membrane potential
PDT: Photodynamic therapy
PTT: Photothermal therapy
CaCl_2_: Calcium chloride
NH_4_HCO_3_: Ammonium bicarbonate
CO_3_^2−^: Carbonate ions
EDX: Energy dispersive X-ray spectroscopy
XPS: X-ray photoelectron spectroscopy
XRD: X-ray diffractometer
FT-IR: Fourier Transform infrared spectrometer
MTT: Methyl thiazolyl tetrazolium
RhB: Rhodamine B
DCFH-DA: 2′,7′-Dichlorofluorescein diacetate
DAPI: 2-(4-Amidinophenyl)-6-indolecarbamidine dihydrochloride
C6: Coumarin 6
TEM: Transmission electron microscopy
UV–vis: Ultraviolet visible
LE: Loading efficiency
LC: Loading content
H&E: Hematoxylin–eosin
TUNEL: TdT-mediated dUTP nick-end labeling
ALT: Alanine aminotransferase
AST: Aspartate aminotransferase
SD: Standard deviation
RBC: Red blood cells
CO_2_: Carbon dioxide
