# Supplementary material for: Nano-modulators with the function of disrupting mitochondrial Ca2+ homeostasis and photothermal conversion for synergistic breast cancer therapy
Source: J Nanobiotechnology. 2023 Dec 4;21:465. doi: 10.1186/s12951-023-02220-7 (PMC10694906; doi:10.1186/s12951-023-02220-7)
Supplement: Supplementary file 1 — Additional file 1. Additional figures. [file 12951_2023_2220_MOESM1_ESM.docx]

**Additional Information**

**Nano-modulators with the function of disrupting mitochondrial Ca^2+^ homeostasis and photothermal conversion for synergistic breast cancer therapy**

*Chenglong Wang* *^a#^, Tao Li ^b#^, Zhen Wang ^a#^, Yao Li ^c^, Yan Liu ^a^, Maochang Xu ^a^, Zongquan Zhang ^a^, Yiping Deng ^a^, Liang Cai ^d*^, Chunxiang Zhang ^c*^, Chunhong Li ^a*^*

^a^ Department of Pharmaceutical Sciences, School of Pharmacy, Southwest Medical University, Luzhou, Sichuan, 646000, China.

^b^ Key Laboratory of Medical Electrophysiology of Ministry of Education, Institute of Cardiovascular Research, Southwest Medical University, Luzhou, Sichuan Province, China.

^c^ Department of Science and Technology, Southwest Medical University.

^d^ Nuclear Medicine Department of the First Affiliated Hospital, Southwest Medical

University, Luzhou, Sichuan, 646000, China.

^e^ The Key Laboratory of Medical Electrophysiology of the Ministry of Education, Southwest Medical University, No.1, Section 1, Xianglin Road, Luzhou, Sichuan, 646000, China.

*Corresponding author: Liang Cai.

E-mail: cllc131420@sina.com

Main address: Nuclear Medicine Department of the First Affiliated Hospital, Southwest Medical University, Luzhou, Sichuan, 646000, China.

*Corresponding author: Chunxiang Zhang.

E-mail: zhangchx999@163.com

Main address: The Key Laboratory of Medical Electrophysiology of the Ministry of Education, Southwest Medical University, No.1, Section 1, Xianglin Road, Luzhou, Sichuan, 646000, People's Republic of China.

*Corresponding author: Chunhong Li.

E-mail: lispringhong@126.com

Main address: Department of Pharmaceutical Sciences, School of Pharmacy, Southwest Medical University, 1-1 Xianglin Road, Luzhou, Sichuan, 646000, People’s Republic of China


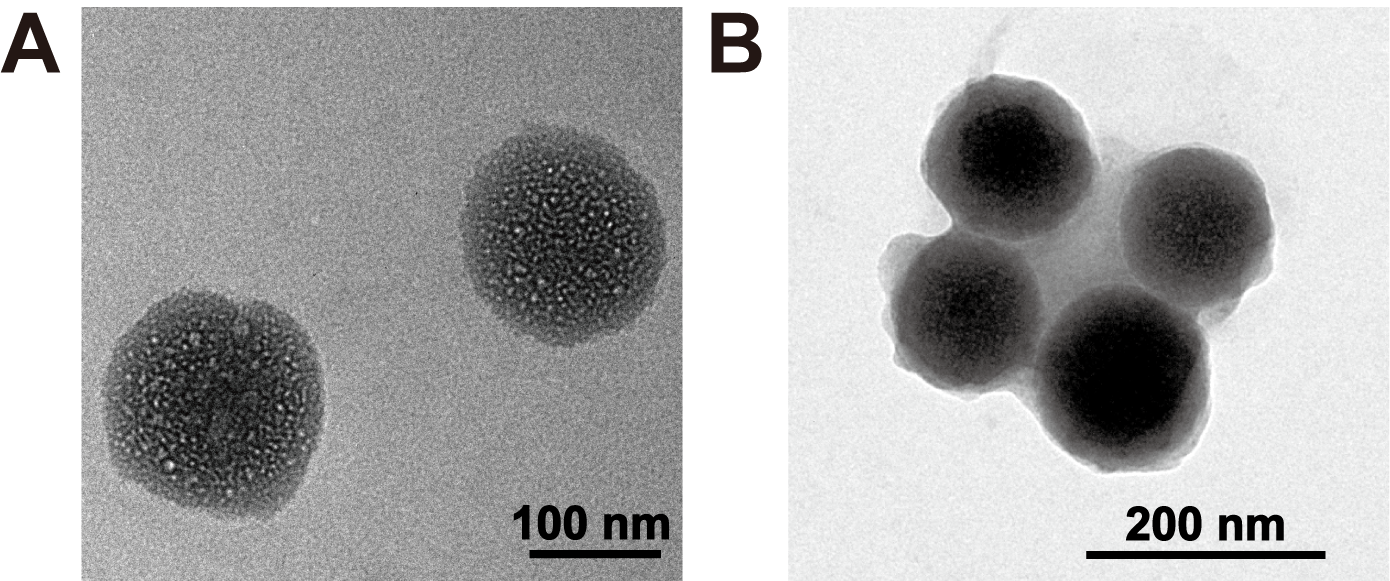


**Fig. S1** (A) The representative TEM image of CCI. Scale bar, 100 nm. (B) The representative TEM image of SCCI. Scale bar, 200 nm.


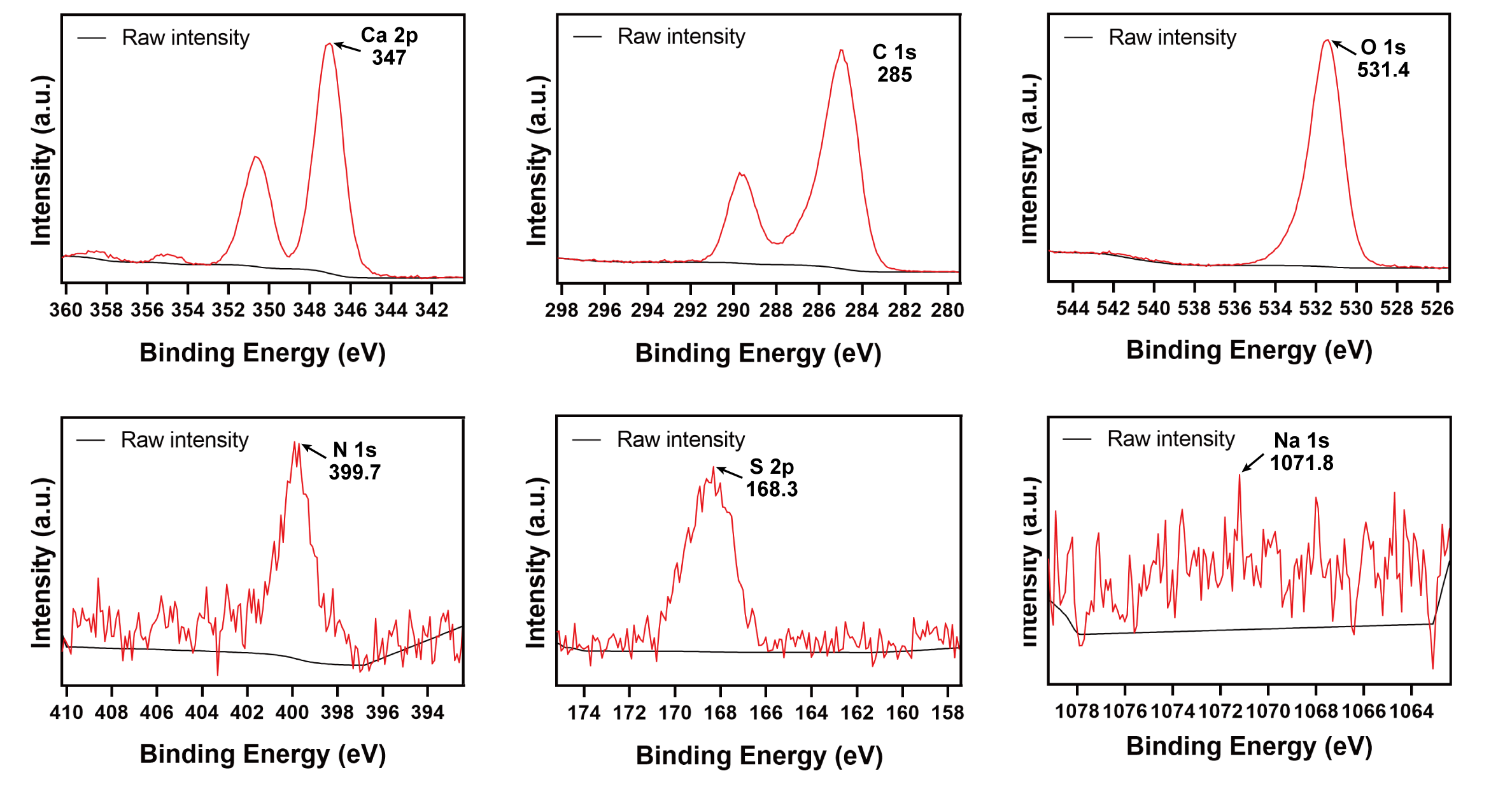


**Fig. S2** XPS analysis of various elements in SCCI.


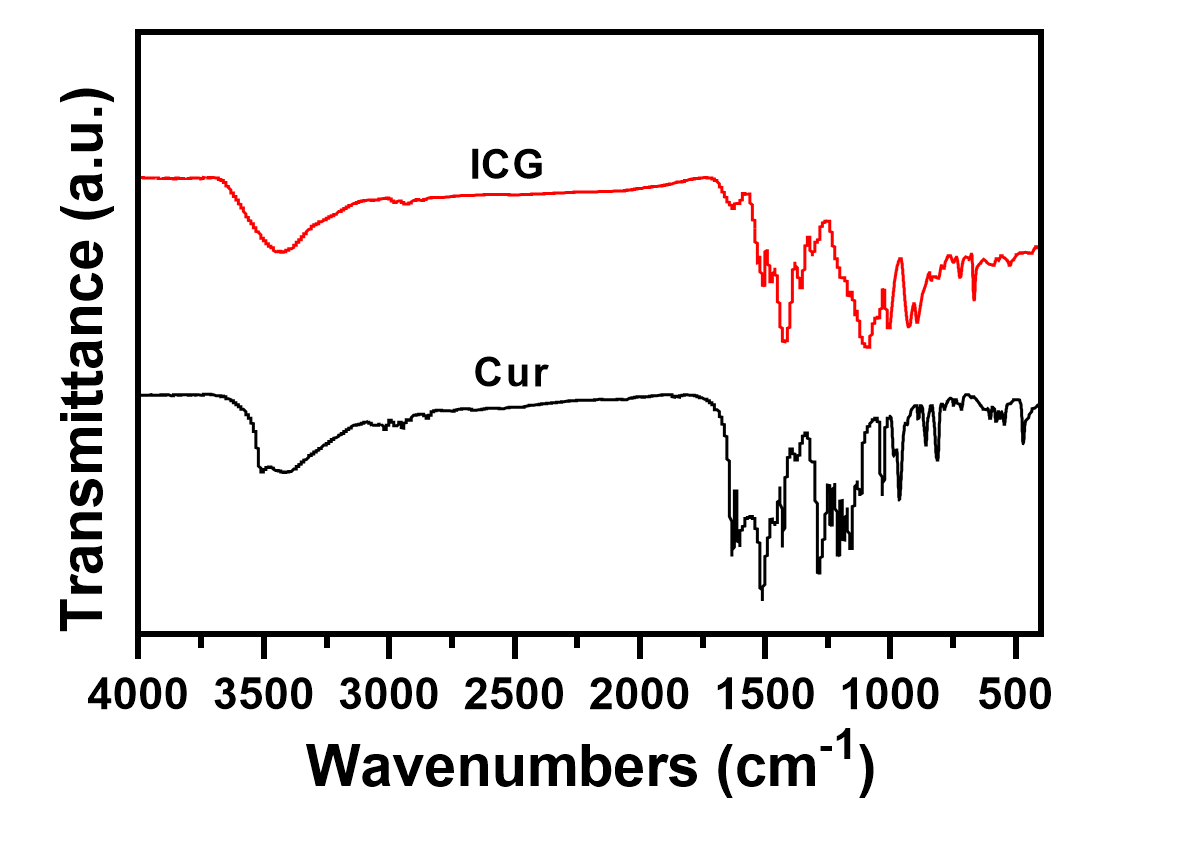


**Fig. S3** FT-IR spectra of Cur and ICG.


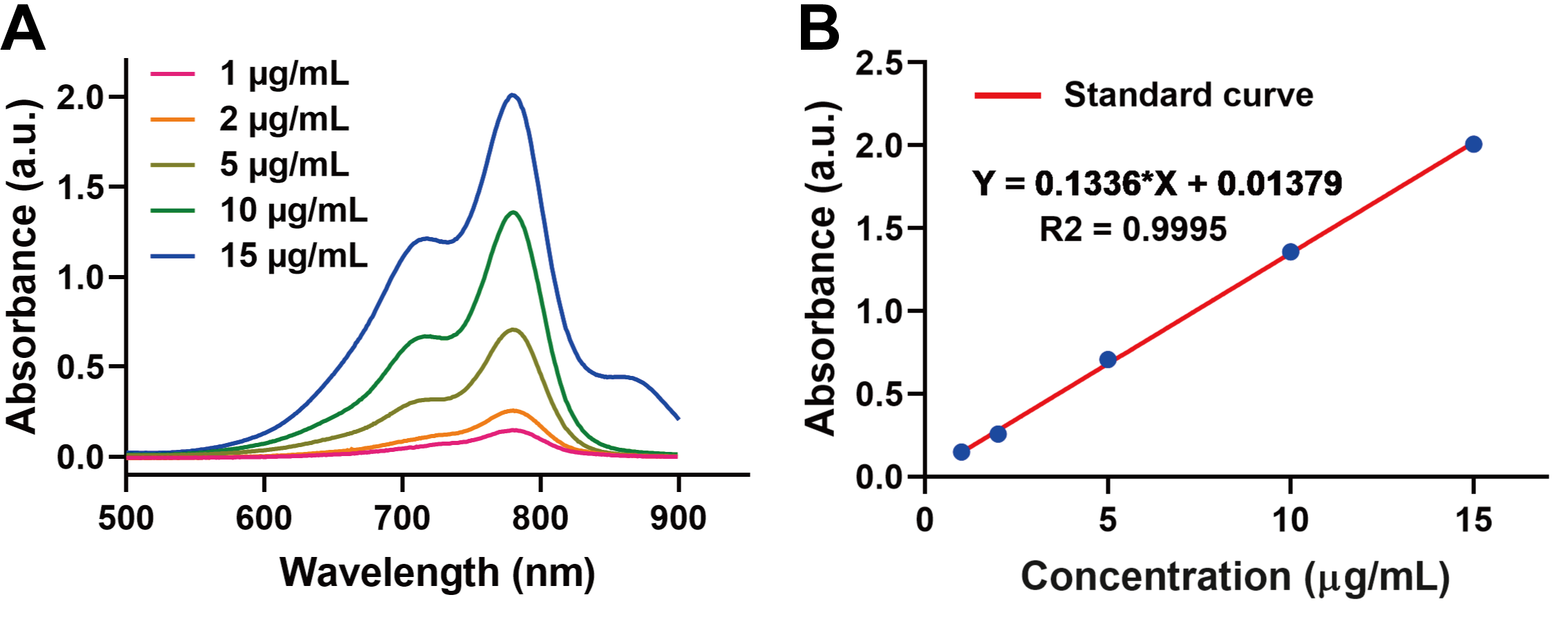


**Fig. S4** Quantitative analysis of ICG. (a) UV-visible absorption spectrum of ICG solution. (b) Standard absorption curve of ICG.


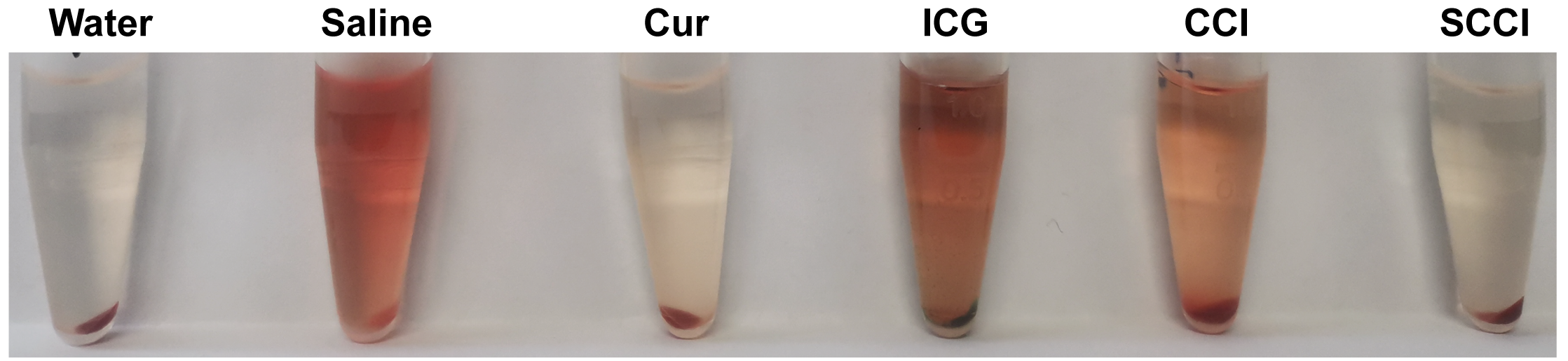


**Fig. S5** Representative images of hemolysis assay when the concentration of Cur, ICG, CCI, and SCCI was 200 µg/mL.


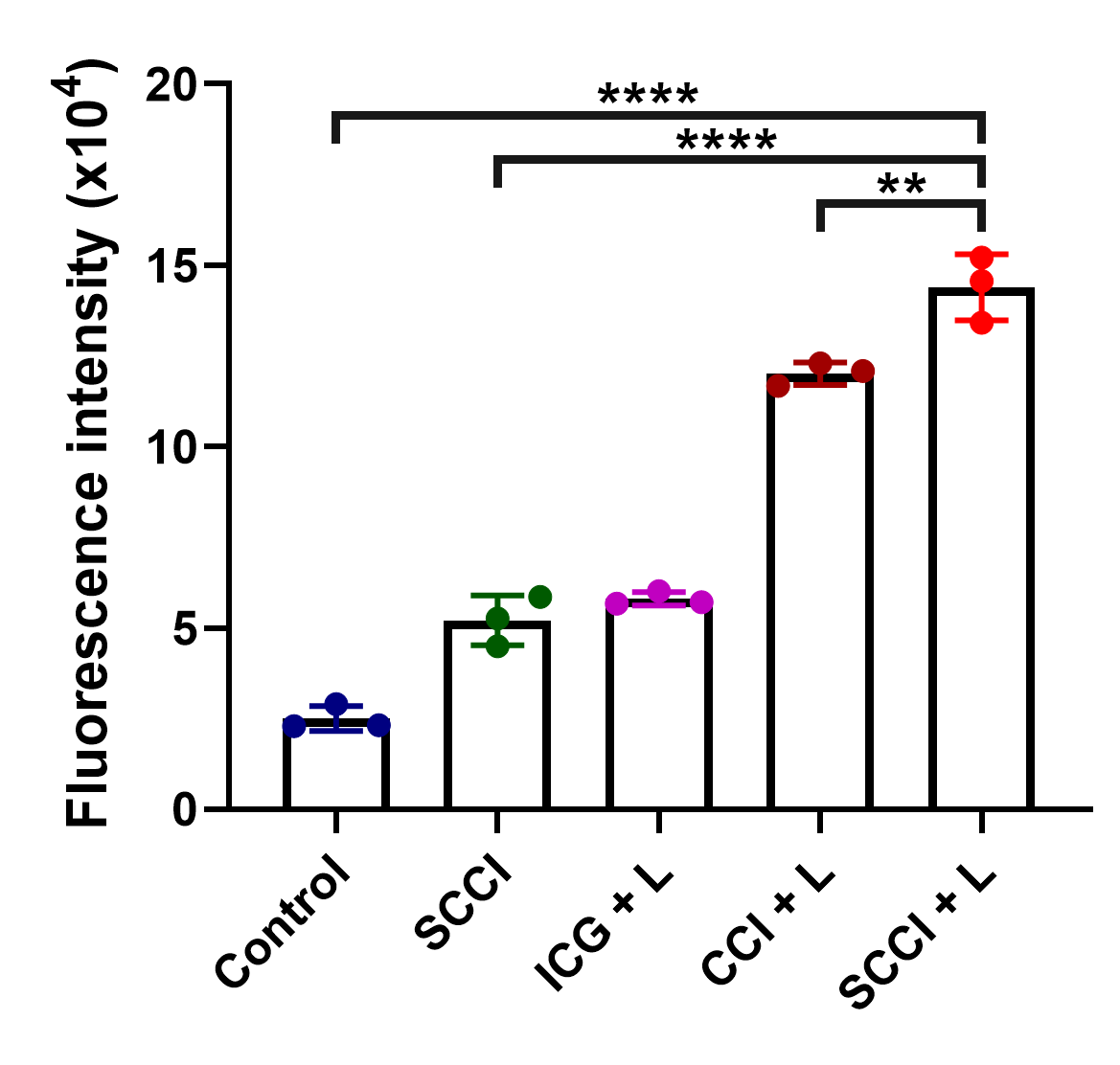


**Fig. S6** The fluorescence intensity generated after incubating DCFH-DA with the cells quantified by flow cytometry. Data are shown as mean ± SD (*n* = 3). *****P* < 0.0001.


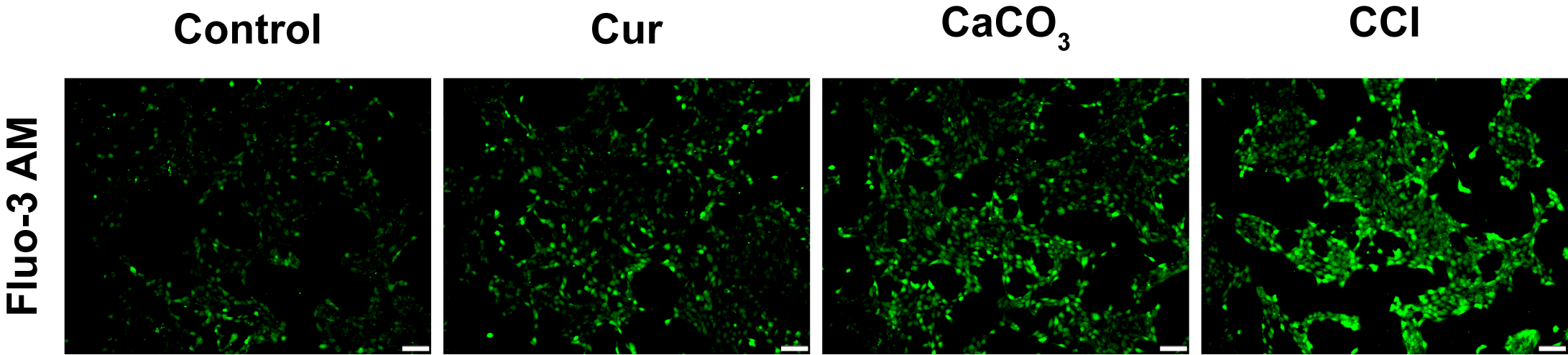


**Fig. S7** Fluorescence microscope images of intracellular Ca^2+^ generation after 6 h treatment with Cur, CaCO_3_ and CCI_._ Scale bar, 100 μm.


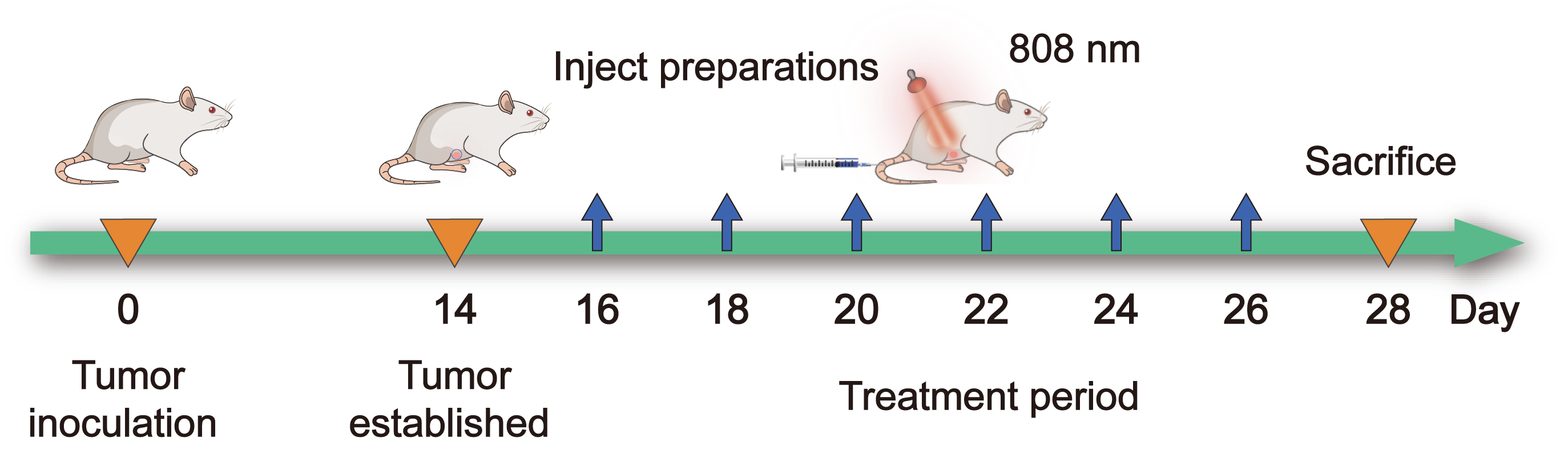


**Fig. S8** Schematic diagram of the establishment and treatment protocol of the 4T1 tumor-bearing mice.


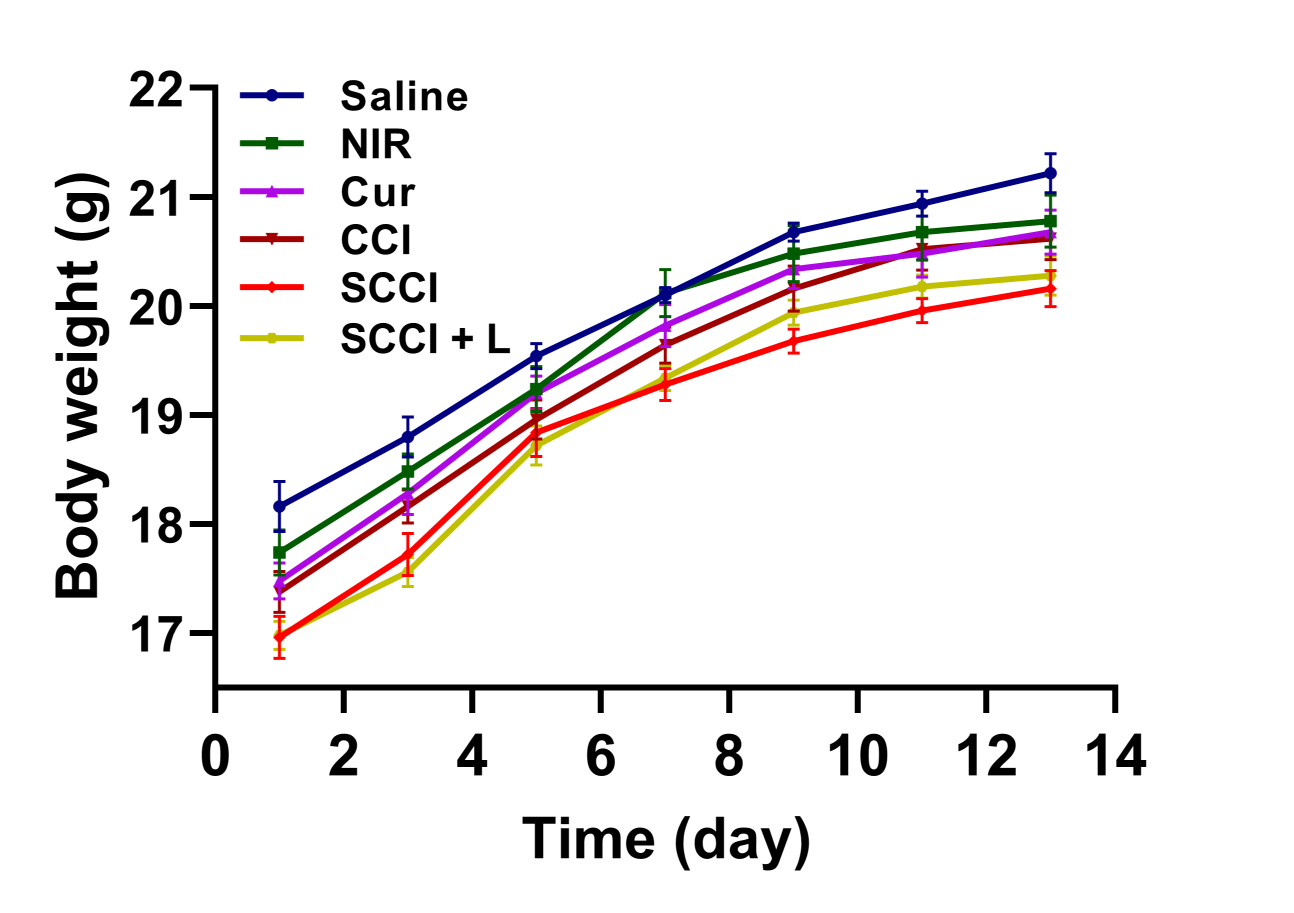


**Fig. S9** Body weight changes in 4T1 tumor-bearing mice after treatment with different formulations. Data are shown as mean ± SD (n =5).


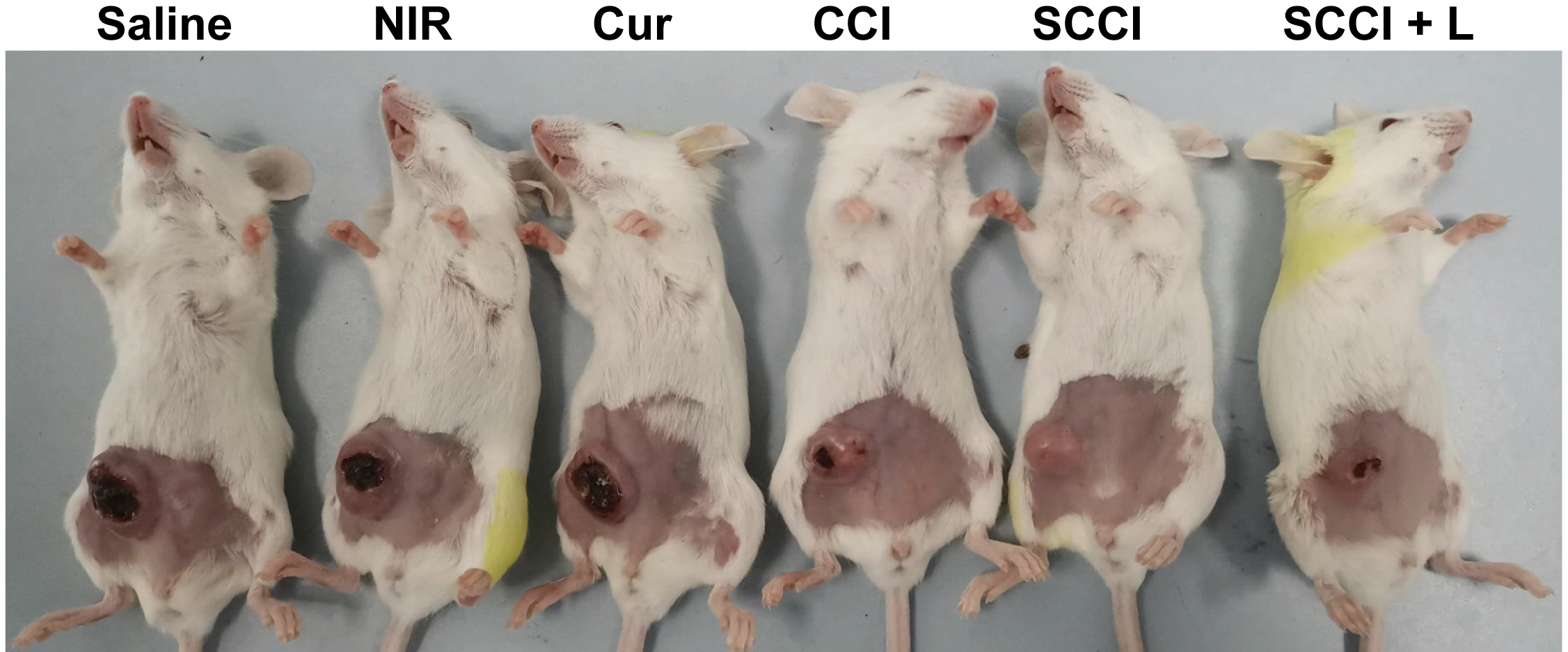


**Fig. S10** Representative treatment plots of tumor-bearing mice, 24 h after the last administration.


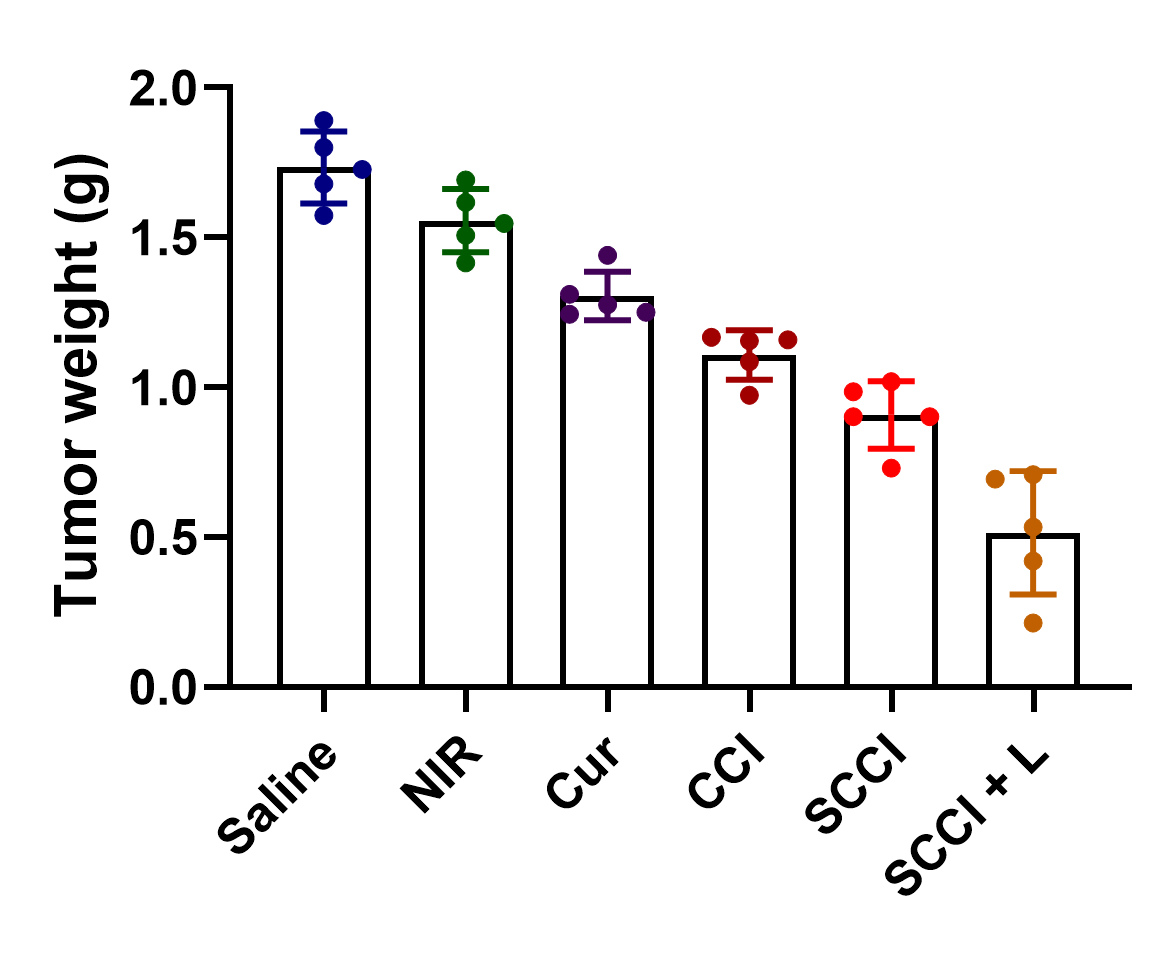


**Fig. S11** Tumor weight variation in 4T1 tumor-bearing mice after treatment with different formulations. Data are shown as mean ± SD (n =5).


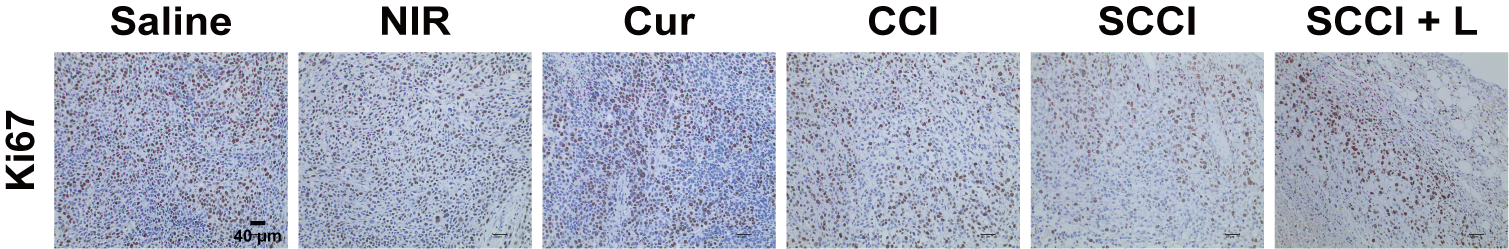


Fig. S12 Representative Ki67 staining images of 4T1 tumors in different treatment groups. Scale bar, 40 μm.


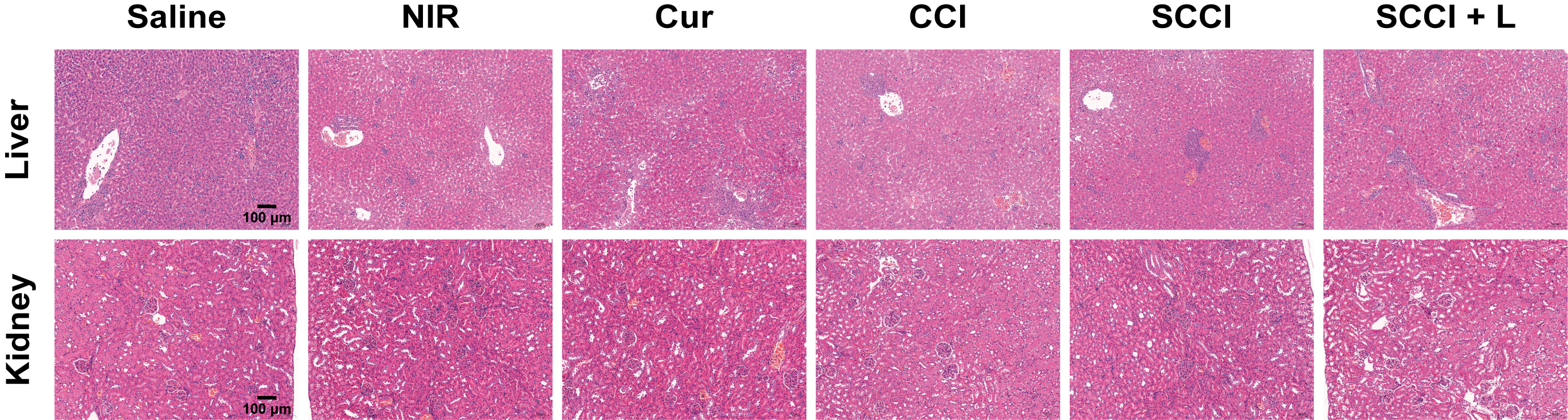


**Fig. S13** Representative H&E staining images of exfoliated hearts and livers in different treatment groups. Scale bar, 100 μm.
